# Precise Characterization of *Bombyx mori Fibroin Heavy Chain* Gene Using Cpf1-Based Enrichment and Oxford Nanopore Technologies

**DOI:** 10.3390/insects12090832

**Published:** 2021-09-16

**Authors:** Wei Lu, Xinhui Lan, Tong Zhang, Hao Sun, Sanyuan Ma, Qingyou Xia

**Affiliations:** 1 State Key Laboratory of Silkworm Genome Biology, Biological Science Research Center, Southwest University, Chongqing 400715, China; luw10@outlook.com (W.L.); lanxinhuii@163.com (X.L.); zt137703197@email.swu.edu.cn (T.Z.); sh040019@email.swu.edu.cn (H.S.); 2 Chongqing Key Laboratory of Sericulture Science, Chongqing Engineering and Technology Research Center for Novel Silk Materials, Chongqing 400715, China

**Keywords:** Cpf1, ONT, *FibH*, methylation

## Abstract

**Simple Summary:**

*Bombyx mori* (*B. mori*), an important economic insect, is famous for its silk. *B. mori* silk is mainly composed of silk fibroin coated with sericin. Among them, the silk fibroin heavy chain protein has the highest content and the largest molecular weight, which is encoded by the silk *fibroin heavy chain* (*FibH*) gene. At present, apart from the complete sequence of the *FibH* of the *B. mori* strain p50T, there are no other reports regarding this protein. This is mainly because the special structure formed by the GC-rich repetitive sequence in *FibH* hinders the amplification of polymerase and the application of Sanger sequencing. Here, the *FibH* sequence of *Dazao*, which has 99.98% similarity to that of p50T, was obtained by means of CEO. As far as we know, this is the first complete *FibH* sequence of the Chinese *B. mori* strain. Additionally, the methylated CG sites in the *FibH* repeat unit were identified.

**Abstract:**

To study the evolution of gene function and a species, it is essential to characterize the tandem repetitive sequences distributed across the genome. Cas9-based enrichment combined with nanopore sequencing is an important technique for targeting repetitive sequences. Cpf1 has low molecular weight, low off-target efficiency, and the same editing efficiency as Cas9. There are numerous studies on enrichment sequencing using Cas9 combined with nanopore, while there are only a few studies on the enrichment sequencing of long and highly repetitive genes using Cpf1. We developed Cpf1-based enrichment combined with ONT sequencing (CEO) to characterize the *B. mori FibH* gene, which is composed of many repeat units with a long and GC-rich sequence up to 17 kb and is not easily amplified by means of a polymerase chain reaction (PCR). CEO has four steps: the dephosphorylation of genomic DNA, the Cpf1 targeted cleavage of *FibH*, adapter ligation, and ONT sequencing. Using CEO, we determined the fine structure of *B. mori* *FibH*, which is 16,845 bp long and includes 12 repetitive domains separated by amorphous regions. Except for the difference of three bases in the intron from the reference gene, the other sequences are identical. Surprisingly, many methylated CG sites were found and distributed unevenly on the *FibH* repeat unit. The CEO we established is an available means to depict highly repetitive genes, but also a supplement to the enrichment method based on Cas9.

## 1. Introduction

Tandem repeat is a common repetitive DNA sequence in eukaryotes, which are arranged consecutively in the genome, and has been called junk or even selfish DNA because most of these DNA sequences cannot encode proteins [1,2]. In fact, these “junk” DNAs are not useless, and they play a very important role in nucleus formation, chromatin rearrangement, tumorigenesis, and gene expression regulation [3,4,5,6,7]. Tandem repeats are mainly located in non-coding regions, such as in the telomeric and centromeric DNA [8,9,10], and a few are located in protein-coding genes, such as those encoding spider silk spidroin [11,12], *B. mori* silk fibroin [13,14,15], disease-related genes [8,16,17]. Variation in the number of repeat units in protein-coding genes determines gene polymorphism and affects the function of the gene. Such genes often have large variations in length and a complex structure, often making it difficult for them to amplify or conduct short-read sequencing [18].

Since the invention of Sanger sequencing in 1977, new sequencing technologies have emerged. Third-generation sequencing not only allows long-read lengths but also provides more information on DNA methylation and is therefore preferred by researchers over other techniques [19,20,21,22]. Third-generation sequencing includes two sequencing platforms, PacBio (PB) and Oxford Nanopore Technologies (ONT). The accuracy of PB is higher; however, ONT allows longer reads [23]. The combination of ONT and PB has breakthrough significance for detecting genomic structural variation and for studying long terminal repeat (LTR) hot spots in large and complex genomes [24]. However, using ONT and PB to assemble a whole genome for only the sites of interest in the reference genome will undoubtedly increase the cost of sequencing and analysis. Enriching or cloning target DNA fragments before ONT sequencing would efficiently solve this problem.

CRISPR/Cas9 is an acquired immune defense system in bacteria and archaea that can be used against invasive viruses and any foreign DNA [25]. Today, a modified CRISPR/Cas9 has been widely used in genome editing in in vivo as well as in in vitro cloning. In addition, CRISPR/Cas9 combined with ONT sequencing has been used to precisely characterize gene duplication and variation. Cpf1 (Cas12a) is a class II CRISPR effector protein that can cleave target DNA under the guidance of a single CRISPR RNA (crRNA) [26,27]. A total of 46 Cpf1 family proteins have been found, of which the functions of 32 members have been analyzed, and only 7 were determined to undergo editing activity in human cells. The well-studied and most used Cpf1 orthologs are *Acidaminococcus sp. BV3L6* (AsCpf1), *Lachnospiraceae bacterium ND2006* (LbCpf1), and *Fransicella novicida U112* (FnCpf1) [26,28,29]. Unlike Cas9, Cpf1 requires a shorter crRNA, which is conducive to the delivery of crRNA libraries using viruses to edit multiple genomes [30]. Cpf1 cuts DNA and produces sticky ends, which facilitate the insertion of new DNA sequences. The cleavage site is far away from its recognition site; therefore, multiple edits in succession are possible [31]. At the same time, Cpf1 has lower off-target activity, which makes Cpf1 a strong competitor of Cas9 [32,33]. Cpf1 has become an efficient and seamless genome and DNA editing tool [34,35,36,37,38]; however, only a few studies have focused on the enrichment of the target region in the genome by Cpf1. In this study, we established Cpf1-based enrichment and ONT sequencing (CEO). CEO includes four steps: the dephosphorylation of gDNA, Cpf1 cleavage, adapter ligation, and ONT sequencing.

Cocoon silk protein is mainly composed of sericin-coated fibroin, which includes silk fibroin heavy chain protein (FibH), silk fibroin light chain protein (FibL), and glycoprotein P25 [39,40]. Among them, FibH has the largest molecular weight and is composed of massive repeat elements. The full length of its coding gene is 16,848 bp, where exon 1 comprises 57 bp, and exon 2 comprises 15,810 bp. Exon 2 is composed of units repeated hundreds of times, and its GC content is as high as 59% [13]. The length of *FibH* may be an important factor affecting the mechanical properties of cocoon silk, but there are only a few studies on this gene, which is mainly because the *FibH* sequence is highly repetitive and not easily amplified by traditional PCR. This study uses the *B. mori* as a model to precisely characterize the sequence of *FibH*, which will provide a reference for the analysis of other large and highly repetitive genes. At the same time, CEO is also an important supplement to the Cas9-based enrichment sequencing method [20,41,42,43,44,45,46,47].

## 2. Materials and Methods

### 2.1. Genomic DNA Extraction

To reduce the cost and to increase the yield of gDNA extraction, we adopted the classical phenol extraction method. Briefly, a *B. mori* pupa ground in liquid nitrogen was incubated in an extraction buffer (1 M Tris-HCl pH 8.0, 0.5 M EDTA pH 8.0, 0.5% SDS) with RNase A at 37 °C for 1 h, and it was then digested with proteinase K (100 µg/mL) at 55 °C overnight to completely degrade the proteins. Next, it was extracted twice with saturated phenol, once with Tris saturated phenol-chloroform-isoamyl alcohol (25:24:1), and once with chloroform. Finally, ethanol was added to the extracted supernatant to precipitate the gDNA. Carotenoids are often precipitated with *B. mori* pupal gDNA. Therefore, to obtain high purity gDNA, we repeated the extraction process again and extracted the gDNA three times with phenol and chloroform. The DNA pellet was resuspended overnight with EB (1 M Tris-HCl pH 8.0, 0.5 M EDTA pH 8.0) at 4 °C, and it was then centrifuged at 10,080 *g* for 10 min at 4 °C to obtain the supernatant and a viscous lower suspension. The former was transparent, while the latter was turbid and viscous. The DNA concentration in the supernatant and the DNA suspension was determined using Nanodrop and Qubit. The supernatant DNA was used for the subsequent experiments.

### 2.2. PCR

The forward and reverse primers (Table S1) were synthesized by Tsingke (Beijing, China) and were dissolved in deionized water to amplify the upstream and downstream regions of the 4 crRNA targeting sites (Supplementary Notes S1–S4). PCR was performed using 160 ng *B. mori* pupal gDNA as DNA templates in a 40 µL reaction mixture containing 20 µL PrimeSTAR Max Premix (2×) (TAKARA, Otsu, Japan, Cat. R045A) and 1.2 µL 10 uM forward and reverse primers. The thermal cycle program was as follows: 98 °C for 4 min, 30 cycles of 98 °C for 10 s, 55 °C for 15 s, 72 °C for 30 s, and 5 min of final extension at 72 °C, using a S1000 Thermal Cycler (Bio-Rad, Hercules, CA, USA). Each PCR product was mixed with 8 µL 5× DNA Loading Buffer with GelRed (Biomed, Beijing, China, EL107-01), separated by 1% agarose gel via electrophoresis, and purified using a gel extraction kit (OMEGA, Biel, Switzerland, Cat. D2500-02) according to the manufacturer’s manual. The purified DNA was immediately cleaved by Cpf1/crRNA or stored at −20 °C.

### 2.3. Cpf1/crRNA Preparation

We mixed 2.5 μL 10 μM crRNA (GenScript, Piscataway Township, NJ, USA) and 2.5 μL 10× cutsmart buffer (NEB, Ipswich, USA, Cat. B7204S) in 20 μL DNase/RNase-free water (Qiagen, Hilden, Germany, Cat. 129115). After heat denaturation at 90 °C for 6 min, we placed it on ice immediately. According to the method in the previous study, the Cpf1 protein expressed *E. coli* BL21(DE3) and was purified [26]. Then 1.4 μL FnCpf1 (17 μM) was added to the above denatured crRNA, mixed well, incubated at 25 °C for 20 min, and placed on ice for later use. Accordingly, four Cpf1/crRNA complexes were prepared, which were next used to cleave the PCR product and the gDNA.

To verify the availability of the four Cpf1/crRNA complexes, the purified DNA was cleaved by the Cpf1/crRNA complex for 1 h at 37 °C and then separated by 1% agarose gel via electrophoresis. The cleavage reaction was composed of 1 µL Cpf1/crRNA complex, 1 µL purified DNA, 1 µL 10× cutsmart buffer, and 7 µL DNase/RNase-free water. The DNA bands were analyzed by ImageJ (Available online: https://imagej.nih.gov/ij/ (accessed on 1 January 2020)) to estimate the cleavage efficiency of the four Cpf1/crRNA complexes.

### 2.4. Nanopore Sequencing Library Preparation

For dephosphorylation, 3 µg DNA was dissolved in 17 µL DNase/RNase-free water, and then 2 µL 10× cutsmart buffer and 1 µL CIP (NEB, Cat. M0290V) were mixed well and incubated at 37 °C for 30 min followed by the thermal inactivation of the enzyme at 80 °C for 5 min. After cooling to 25 °C, 1 μL of 1 mM dNTP (NEB, Cat. N0447S), 0.5 μL of 10 mM dATP (NEB, Cat. N0440S), 5 μL each of the 4 Cpf1/crRNA complexes, 1 μL 10× cutsmart buffer, and 6.5 μL DNase/RNase-free water were added to the system after being cooled to 25 °C and were mixed well. Next, 1 μL Klenow Fragment (3′→5′ exo-) (NEB, Cat. M0212L) was added and mixed well. CRISPR digestion, end repair, and poly-A tail addition were performed at 37 °C. The enzymes were inactivated at 67 °C for 7 min. The reaction solution was purified using 1× Ampure XP magnetic beads (Beckman, Brea, CA, USA, Cat. A63882), washed twice with 75% ethanol, dried, and 62 μL EB was added (QIAGEN, Valencia, CA, USA, Cat. 19086). Next, 60 μL DNA, 5 μL AMX (Nanopore, Oxford, UK, Cat. SQK-LSK109), 10 μL quick T4 DNA ligase (NEB, Cat. E6056L), and 25 μL LNB (Nanopore, Oxford, UK, Cat. SQK-LSK109) were mixed well and incubated at 25 °C for 30 min to add adaptors. The reaction solution was purified using 0.4× Ampure XP magnetic beads, washed twice with LFB (Nanopore, Oxford, UK, Cat. SQK-LSK109), and then dissolved with 26 μL EB (Nanopore, Oxford, UK, Cat. SQK-LSK109). Next, 75 μL SQB (Nanopore, Oxford, UK, Cat. SQK-LSK109) and 51 μL LB (Nanopore, Oxford, UK, Cat. SQK-LSK109) were added to 24 μL DNA and mixed thoroughly. The sequencing was conducted according to the protocols created by Nanopore PromethIon (Nanopore, Oxford, UK, Cat. SQK-LSK109).

### 2.5. Data Analysis

Based on the silkworm reference genome (Available online: http://silkbase.ab.a.u-tokyo.ac.jp/cgi-bin/index.cgi (accessed on 5 November 2019)) [48], the crRNA sequences were designed using the online version of CCTop (Available online: http://crispr.cos.uni-heidelberg.de/ (accessed on 1 December 2019)) [49]. In addition, the selected crRNAs were mapped against the silkworm reference genome with bowtie2 (v2.3.5) [50] to check the degree of sequence specificity. Nanopore raw FAST5 reads were base called used Guppy (Available online: https://pypi.org/project/ont-pyguppy-client-lib/ (accessed on 2 March 2020)) to obtain the original data. The reads with a quality value less than 7 were filtered out, and high-quality data (quality value greater than or equal to 7) were used for subsequent analysis. The ONT Reads were aligned to the silkworm reference genome using Minimap2 (v 2.17-r941) [51], and then samtools (v1.9) [52] was used to extract the reads that were aligned to the target region and the number of reads was counted. Canu (v1.7) [53] was used to assemble the reads that mapped to the target area. After assembly, Medaka software (Available online: https://github.com/nanoporetech/medaka (accessed on 23 March 2020)) was used to align the ONT reads to the assembled sequence for error correction on the assembled sequence to obtain a consensus sequence. Based on the reference genome sequence, nanopolish software (Available online: https://github.com/shiliyan/nanopolish (accessed on 15 April 2020)) was used to detect the methylation of the target region.

## 3. Results

### 3.1. Cpf1-Based Enrichment and ONT Sequencing (CEO) Overview

**Figure 1:**
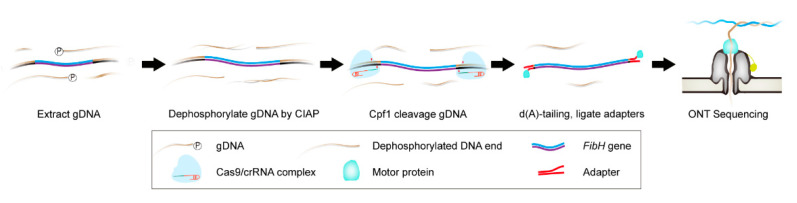
Overview of Cpf1-based enrichment and ONT sequencing (CEO) strategy. CEO mainly includes the dephosphorylation of gDNA, Cpf1 cleavage, adapter ligation, and ONT sequencing. CIAP was used to dephosphorylate the *B. mori* genomic DNA (gDNA), and the target site was released by cleavage with Cpf1/crRNA RNP. Next, the sticky ends were filled in, and the adapters were ligated, and finally, ONT sequencing was performed using PromethION.

To precisely characterize large, highly repetitive, and less complex genes, we established the CEO strategy (Figure 1). The strategy was divided into four steps: dephosphorylation, Cpf1 digestion, adapter ligation, and sequencing analysis. Briefly, *B. mori* gDNA was dephosphorylated with CIAP to block the 5′ end of all the linearized DNA. The dephosphorylated gDNA was then digested with the Cpf1/crRNA complex to release the target region. Then, the sticky ends left by Cpf1 cleavage were filled by a poly-A tail, and the adaptors were ligated. Finally, the complete sequence of the target gene was obtained through ONT sequencing and bioinformatic analysis.

### 3.2. Preparation of High-Quality B. mori gDNA and High Activity crRNA

**Figure 2:**
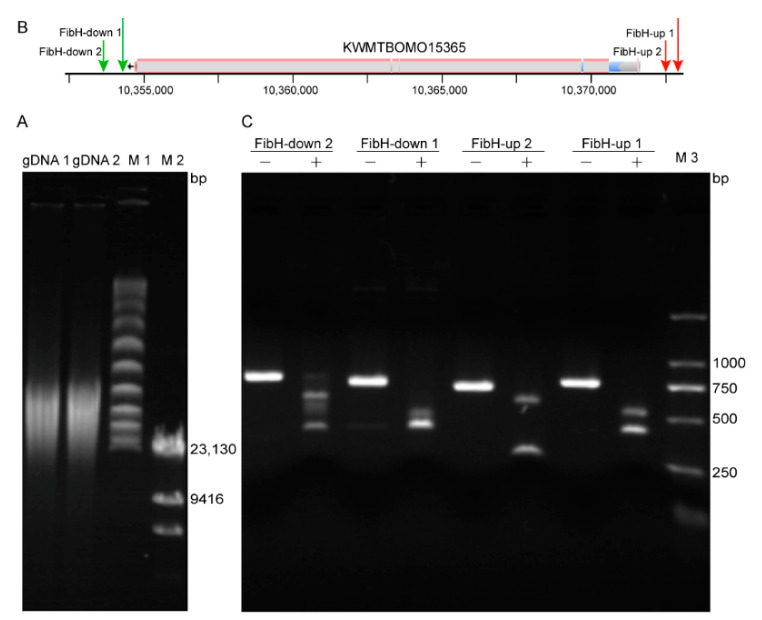
Cleavage efficiency of crRNA. (**A**) The *B. mori* gDNA obtained by phenol-chloroform extraction was separated by pulsed field gel electrophoresis (PFGE). gDNA 1 and gDNA 2 were in the supernatant and the lower suspension, respectively. M1: Lambda PFGE Ladder; M2: λ DNA/*Hind* III (23,130, 9416, 6557, 4361, 2322, 2027, 564 bp). (**B**) The position of crRNA in the *B. mori* genome. Four crRNAs, FibH-up 1, FibH-up 2, FibH-down 1, and FibH-down 2, were located upstream and downstream of the reference gene (KWMTBOMO15365). (**C**) The activity of the four crRNAs in (**B**). The four crRNA-target sequences were obtained by PCR, and the PCR product was cleaved in vitro with the Cpf1/crRNA complex. “+” and “−” indicate the addition and absence of crRNA in the Cpf1 cleavage reaction, respectively. M3: D2000 Marker.

ONT sequencing is known for its long read-length, which is determined by the integrity of the gDNA. To obtain high-quality gDNA, it was extracted using phenol-chloroform from a *B. mori* pupa. Because carotenoids are easily extracted from *B. mori* pupae along with gDNA and affect subsequent experiments, they were therefore removed by repeating the extraction process. Suspension and supernatant DNA were obtained by high-speed centrifugation at 4 °C, and the supernatant DNA was used for subsequent enrichment. The DNA fragments were 23–200 kb (Figure 2A), which spanned the entire *FibH* (KWMTBOMO15365) and met the needs for ONT library construction.

Cpf1 is not only an efficient genome editing tool, but it is also an important method for DNA assembly and cloning [31,34,54,55]. Whether in vivo or in vitro, its cleavage activity is affected by the DNA environment and crRNA sequence [56]. To obtain highly active crRNA, four crRNAs, FibH-up 1, FibH-up 2, FibH-down 1, and FibH-down 2, which target the upstream and downstream of *FibH* (Figure 2B), were designed, and their efficiency score was predicted using CHOPCHOP [57] (Table 1). The residual DNA after the digestion of the Cpf1/crRNA complex was used to determine the cleavage efficiency of the crRNA. Only FibH-down 2 cleaved the purified PCR product incompletely (Figure 2C). Using ImageJ to perform a grayscale analysis of the DNA bands, the cleavage efficiency of FibH-down 2 was about 98%, while the efficiency of the other three was about 100%.

### 3.3. CEO Could Effectively Enrich the Sequence of Interest

**Figure 3:**
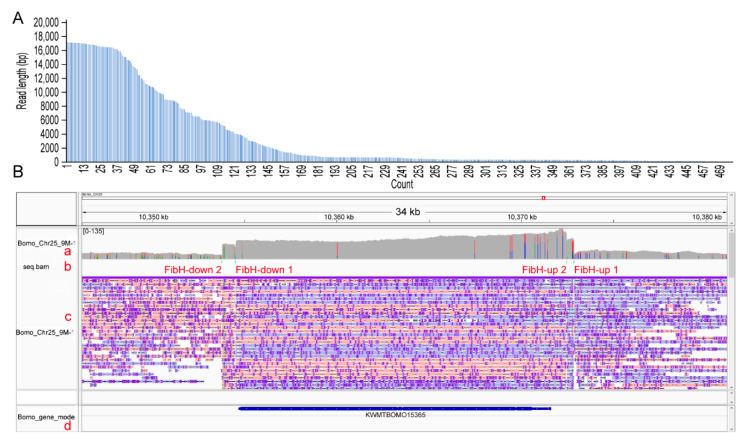
The coverage depth of the target region by Cpf1-based enrichment and ONT sequencing (CEO). (**A**) The length distribution of reads compared to the target region. The horizontal axis is read numbers numbered from high to low according to the read length; the vertical axis is the length of the reads compared to the target region. (**B**) Line a is the depth of read coverage; line b is the position of the crRNA sequence alignment and is marked with the name of the crRNA sequence, and the green line is its position; line c is the read alignment; line d is the gene annotation.

Using CEO, 3,620,449 reads were obtained. There were 349 reads that were mapped to Bomo_Chr25: 10,354,516–10,372,477 target regions, of which 38 reads had a length of 16,000 bp or more (Figure 3A), and some of the reads spanned the reference gene (Figure 3B). ONT sequencing produced 18.21 Gbp of DNA sequencing data, the average depth of the genome was 38×, and the average depth of *FibH* was approximately 87×, with an enrichment fold of 2.29. Previous studies found that the DNA at both ends of the breakpoint could be ligated with an adapter after Cas9 cut the genome. Based on this, Haasteren et al. established the amplification-free integration site sequencing method and applied it to detect the lentiviral vector integration sites in the genome [58]. Similarly, CEO also enriched the upstream (between FibH-up 1 and FibH-up 2) and downstream (between FibH-down 1 and FibH-down 2) region of *FibH*, and the enrichment folds were 2.11 and 1.58, respectively (Table 2). These indicated that CEO could simultaneously perform enrichment analysis on the region of interest in the genome and its upstream and downstream sequences.

### 3.4. CEO Could Characterize the Fine Structure of FibH

**Figure 4:**
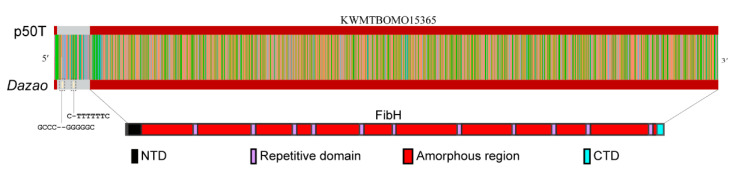
The precise characterization of *FibH* by Cpf1-based enrichment and ONT sequencing (CEO). The upper half of the figure is a comparison between the *FibH* (KWMTBOMO15365) of the Japanese *B. mori* p50T strain and the Chinese *B. mori Dazao* strains using CEO. There were only three base deletions in the intron between the two sequences. The dark red and gray rectangles represent the exon and introns of *FibH*, respectively. The red, yellow, green, and blue lines represent the guanine, cytosine, adenine, and thymine deoxyribonucleotide residues, respectively. The lower half of the figure shows the amino acid sequence deduced from the nucleotide sequence of the *Dazao FibH*. FibH is composed of two terminal non-repetitive domains and a central repetitive core, which includes 12 repetitive domains and 11 separated amorphous regions. Black rectangle, N-terminal domain (NTD); blue rectangle, C-terminal domain (CTD); red rectangle, repeat domain (R); and purple rectangle, amorphous region (A).

ONT reads are long but have random errors. These errors can be corrected by increasing the sequencing depth and deep learning. To verify whether CEO can obtain a high-quality *FibH* consensus sequence (Supplementary Note S5), we assembled the reads aligned to the target region using Canu, and then aligned the ONT reads to the assembled sequence with Medaka to obtain a consensus sequence of 17,988 bp. So far, two *FibH* sequences have been published: one (KWMTBOMO15365) from SilkBase (Available online: http://silkbase.ab.a.u-tokyo.ac.jp (accessed on 2 June 2020)) and the other (AF226688.1) from NCBI. The annotated KWMTBOMO15365 has 4 introns, which is different from AF226688.1. We analyzed all introns and found that, with the exception of intron 1 (971bp) of KWMTBOMO15365, the sequence or partial sequence of the other three introns were identical to the repetitive unit, which implied that KWMTBOMO15365 was probably incorrectly annotated. Therefore, we defined that the reference gene is encoded by two exons (Figure 4). Compared to KWMTBOMO15365, the consensus sequence was identical to the reference sequence, except for 3-base deletion in the intron, with 99.98% identity compared to AF226688.1, with 22 SNPs and 11 gaps/insertions in the consensus sequence, which had 99.17% identity. The difference between KWMTBOMO15365 and AF226688.1 was probably caused by low-quality assembled AF226688.1, but this did not rule out the structural variation in the *FibH* gene between individual silkworms. These results indicated that there were structural variations in the genomes of the Japanese *B. mori* strain p50T and the Chinese strain *Dazao* and also proved that CEO could be used for structural studies of large genes with high repetition and low complexity.

### 3.5. CEO Could Identify Methylation of FibH

**Figure 5:**
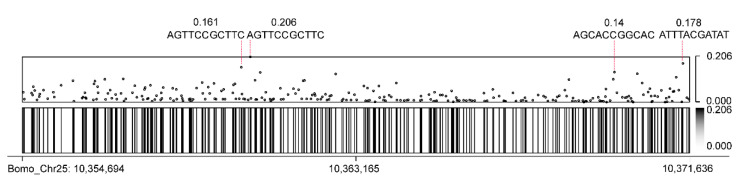
Overview of the distribution of motifs with methylated CG in *FibH*. The dots in the scatter chart and the lines in the heat map represent motifs. The frequency of methylation is marked on the right side of the graph. The sequence and methylation efficiency of the four most methylated sites are marked and indicated by a red dashed line.

As early as 2010, the methylome of the *B. mori* suggested that there are a large number of methylation sites in the *B. mori* genome [59]. Since *FibH* is long and contains many repeat units, the classic bisulfite method cannot detect methylation, and hence, there is no report on *FibH* methylation in *B. mori*. ONT not only has a long read length, but can also recognize modified bases, which is widely used to study base modifications in the genome [22,60,61,62,63]. To identify the presence of base modifications on *FibH*, we performed a methylation analysis on the sequencing data obtained by CEO using Nanopolish. There was a total of 506 CG sites in *FibH*, of which 306 were methylated. Most of these methylated CG sites were located on the repeat units (motifs). The methylation frequency of each motif and their methylation frequency at different positions on the *FibH* gene were counted (Table S2). Among them, motif-1 had the most repetitions, as many as 145, and the total methylation frequency was 2.121, followed by motif-2 and motif-3 with 92 and 65 times, respectively, and their methylation frequencies were 1.782 and 3.251, respectively. Surprisingly, although motif-10 only has three repetitions, its methylation frequency was higher than that of motif-4, and its methylation frequency at Bomo_Chr25: 10,360,481 was as high as 0.206, which was higher than all the other single sites (Figure 5, Table 3). These results indicate that there were abundant methylated cytosines on the repeat units of *FibH* and that the number of repeat units was not correlated with the methylation frequency.

## 4. Discussion

We established an enrichment sequencing method based on Cpf1 combined with ONT for genes with low complexity and high repetition. Using CEO, we described the fine structure and methylation modification of the highly repetitive *FibH* gene. The *FibH* of *Dazao* strain had a 99.98% similarity to the reference gene. With the exceptions of the three base deletions in the intron, their exons were identical. This may be due to differences in the strains. This indicates that *FibH* in different *B. mori* strains or individuals show polymorphism. Meanwhile, the methylation modification on the repetitive sequence of *FibH* was discovered by CEO for the first time. However, when methylation occurs and its effect on the expression of *FibH* are still unknown, which will become the focus of our research in the future.

As we all know, the CRISPR/Cas system is an acquired immune response used by bacteria and archaea to prevent the invasion of foreign DNA, such as plasmids and phages [25]. Among the artificially synthesized CRISPR/Cas systems, the Class 2 system represented by Cpf1 and Cas9 is the most widely used. In addition to our CEO method, other Cas9 nanopore sequencing strategies have also been established, including Negative Enrichment [47], CaBagE [44], FUDGE [64], nCATS [65], and AFIS-Seq [58]. The first two methods are based on the fact that the Cas9 rests on the DNA strand for a short time after cutting the DNA, whereas the principles of the latter three methods are similar to those of our CEO method. They all use the CIAP to seal the DNA ends before cutting the gDNA with the Cas protein. The target site sequences enriched by these methods ranged from 1 kb to 100 kb, including sequence replication genes, but their enrichment efficiency varies. The side-by-side experiments comparing target enrichment with nCATS and CaBagE showed that their average depths ranged from 93× to 322× and 30× to 53×, respectively, and the former was 2.6 to 10.7-fold higher than the latter [44]. The median coverage of the *FibH* gene was 87× via CEO. This difference in coverage is attributed to the sequencer. CaBagE and nCATS use MinION, while the CEO uses PromethIon, which has a higher sequencing throughput. In contrast, nCATS has higher enrichment efficiency than other methods. Among them, the enrichment efficiency of Negative Enrichment and CaBagE is mainly affected by the activity of the crRNA and the degree of cleavage of the unprotected DNA by exonuclease, and the main factor that determines the enrichment efficiency of CEO, FUDGE, nCATS, and AFIS-Seq is crRNA (gRNA) activity and the degree of dephosphorylation of gDNA. It is necessary to compare the enrichment efficiency of these methods under the same conditions to study genes.

In addition to silk protein genes, genes with tandem repeats also include many disease-related genes, such as SAMD12 [66], BRCA1 [67], and VHL [41], which are caused by a duplication of repeats in these genes [9,68]. Understanding the sequence variation of these genes could provide clues for clinical treatment. We believe that our CEO will become an important means of disease-related gene research.

## Figures and Tables

**Table 1:** crRNA activity predicted by CHOPCHOP. Efficiency is the abbreviation of the efficiency score, which is obtained by position-specific scoring matrix or support vector machine based on the current literature. MM0, MM1, MM2, and MM3 represent the number of potential off-targets with 0, 1, 2, and 3 mismatches, respectively.

crRNA	Target Sequence *	Genomic Location	GC (%)	Efficiency	MM0	MM1	MM2	MM3	Size (bp)
FibH-up 1	**TTTA**TGTTACCGGGGTCTAGTGAC	Chr25: 10,372,894–10,372,871	55	60	0	0	0	0	20
FibH-up 2	**TTTA**AGCTTGTTGTACAAAACTGC	Chr25: 10,372,500–10,372,477	40	52	0	0	0	1	20
FibH-down 1	**TTTA**TATGAACCTATTGTAATTTAG	Chr25: 10,354,516–10,354,540	24	59	0	0	0	0	21
FibH-down 2	**TTTG**TACCCTCATACCTCAAAGAAC	Chr25:10,353,778–10,353,802	43	42	0	0	0	0	21

* The bases in bold in the target sequence indicate the PAM required for Cpf1 to recognize DNA.

**Table 2:** Enrichment efficiency of the target region.

Chromosome	Description	Start	End	Average Depth	Enrichment Fold
Bomo_Chr25	downstream sequence	10,353,778	10,354,516	60.05	1.58
Bomo_Chr25	KWMTBOMO15365	10,354,516	10,372,477	87.07	2.29
Bomo_Chr25	upstream sequence	10,372,477	10,372,871	80.12	2.11

**Table 3:** Repeated motif in *FibH*.

Motif Name	Motif Sequence	Motif Repetition	Methylation Frequency *
motif-1	TGCTCCGTATC	145	2.121
motif-2	AGCACCGGCAC	92	1.782
motif-3	AGCTCCGCTTC	65	3.251
motif-4	ATATCCGCCAT	11	0.267
motif-5	TACTCCGTATC	10	0.222
motif-6	TGAACCGGCAC	9	0.123
motif-7	AGCTCCGGCAC	8	0.123
motif-8	TGCTCCGTACC	8	0.166
motif-9	AGAACCGGCAC	3	0.092
motif-10	AGTTCCGCTTC	3	0.438

* Methylation frequency = number of called_sites_methylated/number of called_sites.

## Data Availability

Not applicable.

## Supplementary Materials

The following are available online at https://www.mdpi.com/article/10.3390/insects12090832/s1, Table S1: Primers used in this study, Table S2: Called sites methylated on the reference gene, Note S1: The target sequence of FibH-up1 crRNA, Note S2: The target sequence of FibH-up2 crRNA, Note S3: The target sequence of FibH-down1 crRNA, Note S4: The target sequence of FibH-down2 crRNA, Note S5: Consensus sequence from CEO.
