# Supplementary material for: Precise Characterization of Bombyx mori Fibroin Heavy Chain Gene Using Cpf1-Based Enrichment and Oxford Nanopore Technologies
Source: Insects. 2021 Sep 16;12(9):832. doi: 10.3390/insects12090832 (PMC8467315; doi:10.3390/insects12090832)
Supplement: Supplementary file 1 [file insects-12-00832-s001.zip › insects-1334962-supplementary.pdf]

Supplementary Note S1 | The target sequence of FibH-up1 crRNA

TTTGAATAAGTGCTTAATTGCAAGTAACGTAACAATGTTTTAGGGTTCGGTCCTCAATA
AATTCGACCAATAAACCATACAAATTCTTTAACATTTTTTTAATCTTATACTAGCTGACC
CGGCAGACTTCGTGGTGCCTCAATCGATAAATAAAATACCTATGCTTCTGTATAAAATA
AACATAAAACAAACAAAAGGAATCCGTCCGACGGGAGACACATCAAAGGAAAAAC
ATCTTTTTTATTTTTTTACCTTTTAAACCTTCTCTGGACTTCCACAAATAATTTAAGACCA
AAATTAGCCAAATCGGTCTAGCATTTTCGAGTTTTAGCGAGACTAACGAACAGCAATT
CATTTTTATATACACAGA**TTTATGTTACCGGGGTCTAGTGAC**CTAAACGACTTCAGCT
CTAACACTAGGCTAACTCAGGCTTAGTAGCCTGGTCCTAGTGTTAGATTTGAAGTCGTC
TAATGCAAAGATTATTGGATCTGATGGATCCGTAAGGACGTGTCTAGAGCGTCGACGG
TGACTAGCTCCTGCGTGATCAGGAAAAATGTGGAAAGCTTAACGATTTTGTCACATTTT
ACTTATCACAACTTGTTTTTATAATAATTCGCTTAAATGAGCAGCTATTACTTAATCTCGT
AGTGGTTTTTGACAAAATCAGCTTCTTTAGAACTAAAATATCATTTTTTTCGTAATTTTTT
TAATGAAAAATGCTCTAGTGTTATACCTTTCCAAAATCACCATTAATTAGGTAGTGTTTA
AGCTTGTTGTACAAAACTGCCACACGCATTTTTTTCTCCACTGTAGGTTGTAGTTACG

The DNA fragments amplified by lw-Cpf1-up-F1 and lw-Cpf1-up-R1 primers were used for FibH-up1 cleavage. The bold and underlined letters are the crRNA binding sites.

Supplementary Note S2 | The target sequence of FibH-up2 crRNA

GTCGTCTAATGCAAAGATTATTGGATCTGATGGATCCGTAAGGACGTGTCTAGAGCGTC
GACGGTGACTAGCTCCTGCGTGATCAGGAAAAATGTGGAAAGCTTAACGATTTTGTCA
CATTTTACTTATCACAACTTGTTTTTATAATAATTCGCTTAAATGAGCAGCTATTACTTAA
TCTCGTAGTGGTTTTTGACAAAATCAGCTTCTTTAGAACTAAAATATCATTTTTTTCGTA
ATTTTTTTAATGAAAAATGCTCTAGTGTTATACCTTTCCAAAATCACCATTAATTAGGTA
GTG**TTTAAGCTTGTTGTACAAAACTGC**CACACGCATTTTTTTCTCCACTGTAGGTTGT
AGTTACGCGAAAACAAAATCGTTCTGTGAAAATTCAAACAAAAATATTTTTTCGTAAA
AACACTTATCAATGAGTAAAGTAACAATTCATGAATAATTTCATGTAAAAAAAAAATA
CTAGAAAAGGAATTTTTCATTACGAGATGCTTAAAAATCTGTTTCAAGGTAGAGATTTT
TCGATATTTCGGAAAATTTTGTAAAACTGTAAATCCGTAAAATTTTGCTAAACATATATT
GTGTTGTTTTGGTAAGTATTGACCCAAGCTATCACCTCCTGCAGTATGTCGTGCTAATTA
CTGGACACATTGTATAACAGTTCCACTGTATTGACAATAATAAAACCTCTTCATTGACT
TGAGAATGTCTGGACAGATTTGGCTTTGTATTTTTGATTTACAAATGTTTTTTTGGTGATT
TACCCATCCAAGGCATTCTCCAG

The DNA fragments amplified by lw-Cpf1-up-F2 and lw-Cpf1-up-R2 primers were used for FibH-up2 cleavage. The bold and underlined letters are the crRNA binding sites.

Supplementary Note S3 | The target sequence of FibH-down1 crRNA

AACTGTGGAATTCCTAGAAGACAACTAGTTGTTAAATTCAGAGCACTGCCTTGTGTGA
ATTGCTAATTTTTAATATAAAATAACCCTTGTTTCTTACTTCGTCCTGGATACATCTATGT

TTTTTTTTTCGTTAATAAATGAGAGCATTTAAGTTATTGTTTTTAATTACTTTTTTTTAGAA
AACAGATTTCGGATTTTTTGTATGCATTTTATTTGAATGTACTAATATAATCAATTAATCA
ATGAATTCATTTATTTAAGGGATAACAATAATCCATGAATTCACATGCACATTTAAAAC
AAAA**<u>CTAAATTACAATAGGTTCATATAAA</u>**AACAACAAGTATGCCTTCTCAACTAAGA
ATACTATATTGTTTAAACCGTAAAAAAAGTCATATTTCTGTATATCAAAACACATCTAAT
ATTAAAAAAACAGTCAGCAAGCACTTACAAGTGTGGGCTCGGACAGCAATTACCTGG
TCTCAGGAGACACTTGAAGAACGAGAAGCACGTCTCTCTGTCGATTGCGAGGCTCAT
GCACTATCGCTTGAGTCTGAGACCTTTACTGATAGGGAAATCCGTTTGAGCTCTCAGA
GGGTTCGGACAGCAAAAGCTTGCCCGGTCTCAGGAGACATTAGAAGAACGGGAAGC
ATAACTCAATACCGATCGCGTTTCCATTGAGCCTATGCTTCGTGATAATAATAAATAAA
GCCCAAGGTCAGACGCTAAAAGTTGTGGGTGTAGATAT

The DNA fragments amplified by lw-Cpf1-down-F1 and lw-Cpf1-down-R1 primers were used for FibH-down1 cleavage. The bold and underlined letters are the crRNA binding sites.

Supplementary Note S4 | The target sequence of FibH-down2 crRNA

CCCGGTCTCAGGAGACATTAGAAGAACGGGAAGCATAACTCAATACCGATCGCGTTT
CCATTGAGCCTATGCTTCGTGATAATAATAAATAAAGCCCAAGGTCAGACGCTAAAAG
TTGTGGGTGTAGATATAAGTGTTAACTGCTTTTCACACAGTCAACTCGGTCACGCGGTT
ATGTAGCTCTGTCACATGCTAGCAATCCTAGCAACCTGTATGCTTTGATCCCCGATGGC
TATACGTCTACGTCGTGTATAGAGAAGCTTTACTTTAAATTACATTTTTCTAAATACCTA
AATGACGGTGCTTATAAACCAAAAGATATGTCCTAAAACTGCTGCCCAAAGTCGCTAT
TCCACGCGGACGGAGTCGCGGGCACAGCTAGTAATACATAAAATACCTTGGAACTCG
AAACGGTCTTTGAGATATGGGTC**<u>GTTCTTTGAGGTATGAGGGTACAAA</u>**GAATACAAT
ATGGTAAAGAAAGGACACGAGTATTACGAATCGGGGTAGTCAAGAATTCGAAGAAGG
AGAGACTGTGCAAGCGCAGCTTTAGTGGCGGCTGGAAGAAAATTTCGTAACCGTCTT
AAAGATTCAGCTGATGGAAACACCTTCCGACTAACCCCTTTTATCTGTGCGTTCCACA
ATAAGTTTTTATGAATTGAGATTCCCAAATTTTTCACAGTATCACTGATAGGAATTTGTA
CGCCGTCAAATAAAATAACCGGAAGCTGCGACTATTTAATTTTCGAACACATTCTTGA
ATTACTGACAACAATGTCCTGTGTTTTCGACG

The DNA fragments amplified by lw-Cpf1-down-F2 and lw-Cpf1-down-R2 primers were used for FibH-down2 cleavage. The bold and underlined letters are the crRNA binding sites.

Supplementary Note S5 | Consensus sequence from CEO

CATCAGTTCGGTTCCAACTCTCAAGATGAGAGTCAAAACCTTTGTGATCTTGTGCTGC
GCTCTGCAGGTGAGTTAATTATTTTACTATTATTTCAGAAGGTGGCCAGACGATATCAC
GGGCCACCTGATAATAAGTGGTCGCCAAAACGCACAGATATCGTAAATTGTGCCATTT
GATTTGTCACGCCCGGGGGCTACGGAATAAACTACATTTATTTATTTAAAAAATGAACC
TTAGATTATGTAACTTGTGATTTATTTGCGTCAAAAGTAGGCAAGATGAATCTATGTAA
ATACCTGGGCAGACTTGCAATATCCTATTTCACCGGTAAATCAGCATTGCAATATGCAA
TGCATATTCAACAATATGTAAAACAATTCGTAAAGCATCATTAGAAAATAGACGAAAG

AAATTGCATAAAATTATAACCGCATTATTAATTTATTATGATATCTATTAACAATTGCTAT
TGCCTTTTTTCGCAAATTATAATCATTTTCATAACCTCGAGGTAGCATTCTGTTACATTTT
AATACATTGGTATGTGATTATAACACGAGCTGCCCACTGAGTTTCTCGCCAGATCTTCT
CAGTGGGTCGCGTTACCGATCACGTGATAGATTCTATGAAGCACTGCTCTTGTTAGGGC
TAGTGTTAGCAAATTCTTTCAGGTTGAGTCTGAGAGCTCACCTACCCATCGGAGCGTA
GCTGGAATAGGCTACCAGCTAATAGGTAGGGAAAACAAAGCTCGAAACAAGCTCAA
GTAATAACAACATAATGTGACCATAAAATCTCGTGGTGTATGAGATACAATTATGTACT
TTCCCACAAATGTTTACATAATTAGAATGTTGTTCAACTTGCCTAACGCCCCAGCTAGA
ACATTCAATTATTACTATTACCACTACTAAGGCAGTATGTCCTAACTCGTTCCAGATCAG
CGCTAACTTCGATTGAATGTGCGAAATTTATAGCTCAATATTTTAGCACTTATCGTATTG
ATTTAAGAAAAAATTGTTAACATTTTGTTTCAGTATGTCGCTTATACAAATGCAAACAT
CAATGATTTTGATGAGGACTATTTTGGGAGTGATGTCACTGTCCAAAGTAGTAATACAA
CAGATGAAATAATTAGAGATGCATCTGGGGCAGTTATCGAAGAACAAATTACAACTAA
AAAAATGCAACGGAAAAATAAAAACCATGGAATACTTGGAAAAAATGAAAAAATGA
TCAAGACGTTCGTTATAACCACGGATTCCGACGGTAACGAGTCCATTGTAGAGGAAGA
TGTGCTCATGAAGACACTTTCCGATGGTACTGTTGCTCAAAGTTATGTTGCTGCTGATG
CGGGAGCATATTCTCAGAGCGGGCCATACGTATCAAACAGTGGATACAGCACTCATCA
AGGATATACGAGCGATTTCAGCACTAGTGCTGCAGTCGGTGCAGGAGCTGGTGCAGGT
GCTGCCGCTGGTTCTGGTGCGGGTGCCGGAGCTGGTTATGGAGCTGCTTCTGGTGCTG
GTGCCGGTGCTGGGGCTGGTGCCGGAGCTGGTTATGGAACTGGTGCAGGTGCAGGTG
CCGGAGCTGGTTATGGAGCTGGTGCAGGTGCAGGTGCCGGAGCTGGTTATGGGGCTG
GTGCAGGTGCAGGTGCCGGAGCTGGTTATGGAGCTGGTGCAGGTGCAGGTGCCGGAG
CTGGTTATGGGGCTGGTGCAGGTGCAGGTGCCGGAGCTGGTTATGGAGCTGGTGCGGG
TGCCGGTGCCGGGGCTGGTTATGGAGCTGCCTCTGGTGCTGGTGCTGGCGCTGGGTAC
GGACAAGGAGTAGGAAGCGGAGCTGCTTCTGGAGCTGGTGCAGGTGCAGGAGCAGG
TTCTGCCGCTGGTTCTGGGGCAGGTGCCGGTGCTGGTACCGGTGCTGGTGCAGGTTAC
GGAGCTGGTGCAGGTGCCGGTGCCGGAGCTGGTTATGGAGCTGCCTCTGGTACTGGA
GCAGGTTATGGAGCTGGTGCCGGAGCTGGTTACGGAGGTGCCTCTGGTGCTGGTGCTG
GTGCCGGTGCTGGGGCTGGAGCCGGTGCTGGTGCAGGTTATGGAACTGGCGCTGGAT
ACGGAGCAGGAGCCGGAGCAGGAGCCGGAGCAGGAGCTGGTGCTGGATACGGAGC
AGGAGCTGGTGCTGGATACGGAGCAGGATATGGAGTAGGAGCTGGTGCTGGATACGG
AGCAGGATACGGAGCAGGAGCTGGAAGCGGAGCTGCCTCTGGTGCTGGTTCAGGTGC
CGGTGCTGGTTCAGGTGCCGGTGCTGGTTCAGGTGCCGGTGCTGGTTCAGGTGCCGGT
GCCGGTTCAGGTGCTGGTGCTGGTTCAGGTGCTGGTGCTGGTTCAGGTGCTGGTGCAG
GCTCAGGTGCTGGTGCTGGTTCAGGTACTGGTGCTGGTTCAGGAGCTGGTGCTGGATA
CGGAGCAGGAGCTGGTGCTGGATACGGAGCAGGAGCAGGAAGTGGAGCTGCCTCTG
GTGCCGGTGCTGGTTCAGGTGCAGGTGCTGGTTCAGGTGCTGGTGCTGGTTCAGGTGC
TGGTGCTGGTTCAGGTGCTGGTGCTGGTTCAGGAGCTGGTGCTGGATACGGAGCAGGA
GCTGGTGCTGGATACGGAGCAGGAGCTGGTGCTGGATACGGAGCAGGAGCTGGCGTT
GGATACGGAGCAGGAGCTGGAAGCGGAGCTGCCTCTGGTGCTGGTGCTGGTTCAGGA
GCCGGTGCTGGTTCAGGTGCTGGTGCTGGTTCAGGTGCTGGTGCTGGTTCAGGTGCTG
GTGCTGGTTCAGGTGCCGGTGCTGGTTCAGGTGCTGGTGCTGGTTCAGGAGCTGGTGC
TGGTTCAGGTGCTGGTGCTGGTTCAGGAGCTGGAGTTGGATACGGAGCAGGAGTTGGT
GCTGGATACGGAGCAGGATATGGAGCAGGAGCTGGTGCTGGATACGGAGCAGGAGC

AGGAAGCGGAGCTGCCTCTGGTGCTGGTGCCGGTGCTGGAGCTGGTGCAGGAACAGG
CTCTTCTGGATTTGGACCATATGTAGCAAATGGCGGATATAGCAGAAGTGATGGCTACG
AATACGCTTGGTCGTCTGACTTTGGAACTGGAAGCGGAGCTGGTGCTGGTTCAGGTGC
TGGTGCTGGTTCAGGTGCTGGCGCTGGCTCAGGTGCTGGTGCTGGTTCAGGTGCTGGT
GCTGGTTCAGGAGCTGGAGCTGGATACGGAGCAGGAGTTGGTGTTGGATACGGAGCA
GGATATGGAGCAGGAGCTGGTGCTGGATACGGAGCAGGAGCAGGAAGCGGAGCTGC
CTCTGGTGCCGGTGCTGGTTCAGGTGCTGGTGCTGGTTCAGGTGCCGGTGCTGGTTCA
GGTGCTGGTGCTGGTTCAGGTGCCGGTGCTGGTTCAGGTGCTGGTGCTGGCTCAGGTG
CCGGTGCTGGTTCAGGTGCTGGTGCTGGTTCAGGTGCCGGTGCTGGTTCAGGTGCTGG
TGCTGGTTCAGGTGCTGGTGTTGGCTCAGGTGCTGGTGCTGGTTCAGGTGCTGGTGCTG
GTTCAGGAGCTGGTGCTGGATACGGAGCAGGAGCTGGTGCTGGATACGGAGCAGGAG
CTGGTGCTGGATACGGAGCAGGATATGGAGTAGGAGCTGGTGCTGGATACGGAGCAG
GAGCTGGAAGCGGAGCTGCCTCTGGTGCTGGTGCTGGTTCAGGTGCCGGTGCTGGTTC
AGGTGCTGGTGCTGGTTCAGGTGCTGGTGCTGGTTCAGGAGCTGGAGCTGGTTCAGGT
GCTGGTGCTGGTTCAGGTACTGGTGCAGGCTCAGGTGCTGGTGCTGGTTCAGGATCTG
GAGCTGGATACGGAGCAGGAGTTGGTGTTGGATACGGAGCAGGATATGGAGCAGGAG
CTGGTGCTGGATACGGAGCAGGAGCAGGAAGCGGAGCTGCCTCAGGTGCCGGTGCTG
GTTCAGGTGCTGGTGCTGGTTCAGGTGCCGGTGCTGGTTCAGGTGCTGGTGCTGGTTC
AGGTGCTGGTGCTGGTTCAGGTGCTGGTGCTGGTTCAGGTGCTGGTGCTGGTTCAGGT
GCTGGTGCTGGTTCAGGTGCTGGTGCTGGCTCAGGTGCTGGAGCTGGTTCAGGTGCTG
GTGCTGGTTCAGGAGCTGGAGCTGGATACGGAGCAGGAGTTGGTGCTGGATACGGAG
CAGGATATGGAGCAGGAGCTGGTGCTGGATACGGAGCAGGAGCTGGAAGCGGAGCT
GCCTCTGGTGCTGGTTCAGGTGCCGGTGCTGGTTCAGGTGCCGGTGCTGGTGCTGGTT
CAGGTGCTGGTGCTGGTTCAGGTGCTGGTGCTGGTTCAGGTGCTGGTGCTGGCTCAGG
TGCTGGAGCTGGTTCAGGTGCTGGTGCTGGTTCAGGAGCTGGAGCTGGATACGGAGC
AGGAGTTGGTGCTGGATACGGAGCAGGATATGGAGCAGGAGCTGGTGCTGGATACGG
AGCAGGAGCAGGAAGCGGAGCTGCCTCTGGTGCCGGTGCTGGTTCAGGTGCTGGAGC
TGGTTCAGGTGCCGGTGCTGGTTCAGGTGCTGGTGCTGGTTCAGGAGCTGGTGCTGGT
TCAGGAGCTGGTGCTGGTTCAGGTGCTGGTGCTGGTTCAGGAGCTGGAGTTGGATACG
GAGCAGGATATGGAGCAGGAGCTGGTGCTGGATACGGAGCAGGAGCAGGAAGCGGA
GCTGCCTCTGGTGCTGGTGCCGGTGCTGGAGCTGGTGCAGGAACAGGCTCTTCTGGAT
TTGGACCATATGTAGCACATGGCGGATATAGCGGCTACGAATACGCTTGGTCGTCAGA
ATCTGACTTTGGAACTGGAAGCGGAGCTGGTGCTGGTTCAGGTGCTGGTGCTGGTTCA
GGTGCTGGTGCTGGCTCAGGTGCTGGTGCTGGTTCAGGAGCTGGATACGGAGCAGGA
GTTGGTGCTGGATACGGAGCAGGATATGGAGCAGGAGCTGGTGCTGGATACGGAGCA
GGAGCAGGAAGCGGAGCTGGCTCAGGTGCTGGTGCTGGTTCAGGAGCTGGAGCTGGT
TCAGGTGCCGGTGCTGGTTCAGGTGCTGGTGCTGGTTCAGGAGCTGGTGCTGGTTCAG
GTGCTGGTGCTGGTTCAGGAGCTGGTGCTGGTTCAGGTGCCGGTGCTGGTTCAGGTGC
TGGTGCTGGATACGGAGCAGGATATGGAGCAGGAGCTGGTGCTGGATACGGAGCAGG
AGCAGGAAGCGGAGCTGGCTCAGGTGCTGGTGCTGGTTCAGGTGCTGGTGCTGGTTC
AGGAGCTGGAGCTGGTTCAGGTGCCGGTGCTGGTTCAGGTGCTGGTGCTGGTTCAGGT
GCTGGTGCTGGCTCAGGTGCTGGTGCTGGTTCAGGAGCTGGAGCTGGATACGGAGCA
GGAGTTGGTGCTGGATACGGAGCAGGATATGGAGCAGGAGCTGGTGCTGGATACGGA
GCAGGAGCAGGAAGCGGAGCTGGCTCAGGTGCTGGTGCTGGTTCAGGAGCTGGAGC

TGGTTCAGGTGCTGGTGCTGGTTCAGGTGCCGGTGTTGGTTCAGGTGCTGGTGCTGGTT
CAGGAGCTGGTGCTGGTTCAGGTGCCGGTGCTGGTTCAGGTGCTGGTGCTGGATACGG
AGCAGGATATGGAGCAGGAGCTGGTGCTGGATACGGAGCAGGAGCAGGAAGCGGAG
CTGGCTCAGGTGCTGGTGCTGGTTCAGGTGCTGGTGCTGGTTCAGGAGCTGGAGCTGG
TTCAGGTGCCGGTGCTGGTTCAGGTGCTGGTGCTGGTTCAGGAGCTGGTGCTGGTTCA
GGTGCTGGTGCTGGTTCAGGAGCTGGAGTTGGATACGGAGCAGGAGTTGGTGCTGGA
TACGGAGCAGGATATGGAGCAGGAGCTGGTGCTGGATACGGAGCAGGAGCAGGAAG
CGGAGCTGCCTCTGGTGCTGGTGCCGGTGCTGGAGCTGGTGCAGGAACAGGCTCTTCT
GGATTTGGACCATATGTAGCAAATGGCGGATATAGCGGCTACGAATACGCTTGGTCGTC
AGAATCTGACTTTGGAACTGGAAGCGGAGCTGGTGCTGGTTCAGGTGCTGGTGCTGGT
TCAGGTGCTGGTGCTGGCTCAGGTGCTGGTGCTGGTTCAGGAGCTGGAGCTGGATACG
GAGCAGGATATGGAGCAGGAGCTGGTGCTGGATACGGAGCAGGAGCAGGAAGCGGA
GCTGGCTCAGGTGCTGGTGCTGGTTCAGGAGCTGGAGCTGGTTCAGGTGCCGGTGCTG
GTTCAGGTGCTGGTGCTGGTTCAGGTGCCGGTGCTGGTTCAGGTGCTGGTGCTGGTTC
AGGAGCTGGTGCTGGTTCAGGTGCCGGTTCTGGTTCAGGAGCTGGTGCTGGTTCAGGT
GCTGGTGCTGGTTCAGGAGCTGGAGCTGGATACGGAGCAGGAGTTGGTGCTGGATAC
GGAGTAGGATATGGAGCAGGAGCTGGTGCTGGATACGGAGCAGGAGCAGGAAGCGG
AGCTGCCTCTGGTGCTGGTGCCGGTGCTGGAGCTGGTGCAGGAACAGGCTCTTCTGGA
TTTGGACCATATGTAGCACATGGCGGATATAGCGGCTACGAATACGCTTGGTCGTCAGA
ATCTGACTTTGGAACTGGAAGCGGAGCTGGTGCTGGTTCAGGTGCTGGTGCTGGTTCA
GGTGCTGGTGCTGGCTCAGGTGCTGGTGCTGGTTCAGGAGCTGGTGCTGGTTCAGGTG
CTGGTGCTGGTTCAGGAGCTGGAGCTGGATACGGAGCAGGAGTTGGTGCTGGATACG
GAGCAGGATATGGAGCAGGAGCTGGTGCTGGATACGGAGCTGGAGCAGGAAGCGGA
GCTGCCTCTGGTGCCGGTGCTGGTTCAGGTGCTGGTGCTGGTTCAGGTGCCGGTGCTG
GTTCAGGTGCTGGTGCTGGTTCAGGTGCTGGTGCTGGTTCAGGTGCTGGTGCTGGTTC
AGGTGCTGGTGCTGGTTCAGGAGCTGGTGCTGGTTCAGGAGCTGGTGCTGGTTCAGGT
GCCGGTGCTGGTTCAGGAGCTGGAGCAGGATATGGAGCAGGAGCTGGTGCTGGATAC
GGAGCAGGAGCAGGAAGCGGAGCTGGCTCAGGTGCTGGTGCTGGTTCAGGTGCTGGT
GCTGGTTCAGGAGCTGGAGCTGGTTCAGGTGCCGGTGCTGGTTCAGGTGCTGGTGCTG
GTTCAGGTGCCGGTTCTGGTTCAGGAGCTGGTGCTGGTTCAGGTGCTGGTGCTGGTTC
AGGAGCTGGAGCTGGATACGGAGCAGGAGTTGGTGCTGGATACGGAGCAGGATATGG
AGCAGGAGCTGGTGCTGGATACGGAGCAGGAGCAGGAAGCGGAGCTGGCTCAGGTG
CCGGTGCTGGTTCAGGAGCTGGAGCAGGATATGGAGCAGGAGCTGGTGCTGGATACG
GAGCAGGATATGGAGCAGGAGCTGGTGCTGGATACGGAGCAGGAGCAGGAAGCGGA
GCTGGCTCAGGTGCTGGTGCTGGTTCAGGTGCTGGTGCTGGTTCAGGTGCTGGTGCTG
GTTCAGGAGCTGGAGCTGGTTCAGGTGCCGGTGCTGGTTCAGGTGCTGGTGCTGGTTC
AGGTGCCGGTTCTGGTTCAGGTGCTGGTGCTGGTTCAGGAGCTGGAGCTGGTTCAGGT
GCCGGTGCTGGTTCAGGTGCTGGTGCTGGTTCAGGTGCTGGTGCTGGTTCAGGAGCTG
GAGCTGGATACGGAGCAGGAGTTGGTGCTGGATACGGAGCAGGATATGGAGCAGGAG
CTGGTGCTGGATACGGAGCAGGAGCAGGAAGCGGTGCTGGTTCAGGTGCTGGTGCTG
GTTCAGGTGCTGGTGCTGGTTCAGGTGCTGGTGCTGGTTCAGGAGCTGGTGCTGGATA
TGGAGCTGGATACGGAGCAGGAGCTGGAAGCGGAGCTGCCTCTGGTGCTGGTGCCGG
TGCTGGAGCTGGTGCAGGAACAGGCTCTTCTGGATTTGGACCATATGTAGCACATGGC
GGATATAGCGGCTACGAATACGCTTGGTCGTCAGAATCTGACTTTGGAACTGGAAGCG

GAGCTGGTGCTGGTTCAGGTGCTGGTGCTGGCGCAGGTGCTGGTGCTGGTTCAGGAGC
TGGAGCTGGATACGGAGCAGGAGTTGGTGCTGGATACGGAGCAGGATATGGAGCAGG
AGCTGGTGCTGGATACGGAGCAGGAGCAGGAAGCGGAACTGGCTCAGGTGCTGGTG
CTGGTTCAGGAGCTGGAGCTGGATACGGAGCAGGAGTTGGTGCTGGATACGGAGCAG
GAGCAGGAAGCGGAGCTGCCTTTGGTGCCGGTGCTGGTGCTGGTGCTGGTTCAGGTG
CCGGTGCTGGTTCAGGTGCTGGTGCTGGTTCAGGTGCTGGTGCTGGTTCAGGTGCTGG
TGCTGGTTCAGGAGCTGGTGCTGGATACGGAGCAGGGTACGGAGCAGGAGTTGGTGC
TGGATACGGAGCAGGAGCTGGAAGCGGAGCTGCCTCTGGTGCCGGTGCTGGTTCAGG
TGCTGGTGCTGGTTCAGGTGCCGGTGCTGGTTCAGGTGCTGGTGCTGGCTCAGGTGCT
GGTGCTGGTTCAGGAGCTGGAGCTGGATACGGAGCAGGAGTTGGTGCTGGATACGGA
GCAGGATATGGAGCAGGAGCTGGTGCTGGATACGGAGCTGGAGCAGGAAGCGGAGC
TGCCTCTGGTGCCGGTGCTGGTTCAGGTGCTGGTGCTGGTGCTGGTTCAGGTGCCGGT
GCTGGTTCAGGTGCTGGTGCTGGTTCAGGTGCTGGTGCTGGTTCAGGTGCTGGTTCAG
GTGCTGGTGCTGGTTCAGGTGCCGGTGCTGGTTCAGGAGCTGGTGCTGGATACGGAGC
AGGAGCAGGAAGCGGAGCTGCCTCTGGTGCTGGTGCCGGTGCTGGAGCTGGTGCAGG
AACAGGCTCTTCTGGATTTGGACCATATGTAGCAAATGGCGGATATAGCGGCTACGAA
TACGCTTGGTCGTCAGAATCTGACTTTGGAACTGGAAGCGGAGCTGGTGCTGGTTCAG
GTGCTGGTGCTGGTTCAGGTGCTGGTGCTGGCTCAGGTGCTGGTGCTGGTTCAGGAGC
TGGAGCTGGATACAGAGCAGGAGTTGGTGCTGGATACGGAGCAGGATATGGAGCAGG
AGCTGGTGCTGGATACGGAGCAGGAGCAGGAAGCGGAGCTGGCTCAGGTGCTGGTG
CTGGTTCAGGAGCTGGAGCTGGTTCAGGTGCCGGTGCTGGTTCAGGTGCTGGTGCTGG
TTCAGGTGCCGGTGCTGGTTCAGGTGCTGGGGCTGGTTCAGGAGCTGGTGCTGGATAC
GGAGCAGGAGCAGGAAGCGGAGCTGCCTCTGGTGCCGGTGCTGGTTCAGGTGCTGGT
GCTGGTTCAGGTGCCGGTGCTGGTTCAGGTGCTGGTGCTGGTTCAGGAGCTGGTGCTG
GTTCAGGTGCTGGTGCTGGTTCAGGAGCTGGAGCTGGATACGGAGCAGGAGTTGGTG
CTGGATACGGAGTAGGATATGGAGCAGGAGCTGGTGCTGGATACGGAGCAGGAGCAG
GAAGCGGAGCTGGCTCAGGTGCTGGTGCTGGTTCAGGTGCTGGTGCTGGTTCAGGTGC
CGGTGCTGGTTCAGGTGCTGGTGCTGGTTCAGGTGCCGGTTCAGGTGCTGGTGCTGGT
TCAGGAGCTGGTGCTGGTTCAGGTGCTGGTGCTGGTTCAGGTGCTGGTTCAGGTGCTG
GTGCTGGCTCAGGTGCTGGTGCTGGATACGGAGTAGGATATGGAGCAGGAGCTGGTG
CTGGATACGGAGCAGGAGCAGGAAGCGGAGCTGGCTCAGGTGCTGGTGCTGGGTCA
GGTGCCGGTGCTGGTTCAGGTGCTGGTGCTGGTTCAGGTGCCGGTTCAGGTGCTGGTG
CTGGTTCAGGAGCTGGTGCTGGTTCAGGTGCTGGTGCTGGTTCAGGAGCTGGAGCTGG
ATACGGAGCAGGAGTTGGTGCTGGATACGGAGTAGGATATGGAGCAGGAGCTGGTGC
TGGATACGGAGCAGGAGCAGGAAGCGGAGCTGGCTCAGGTGCTGGTGCTGGTTCAGG
TGCTGGTGCTGGTTCAGGTGCCGGTGCTGGTTCAGGTGCTGGTGCTGGTTCAGGAGCT
GGTGCTGGTTCAGGTGCCGGTGCTGGTTCAGGTGCTGGTGCTGGTTCAGGTGCCGGTT
CAGGTGCTGGTGCTGGTTCAGGAGCTGGTGCTGGTTCAGGTGCCGGTGCTGGTTCAGG
TGCTGGTGCTGGTTCAGGTGCCGGTTCAGGTGCTGGTGCTGGTTCAGGAGCTGGTGCT
GGTTCAGGTGCTGGTGCTGGTTCAGGAGCTGGAGCTGGATACGGAGCAGGAGTTGGT
GCTGGATACGGAGTAGGATATGGAGCAGGAGTTGGTGCTGGATACGGAGCAGGAGCA
GGAAGCGGAGCTGCCTCTGGTGCCGGTGCTGGTTCAGGTGCTGGTGCTGGTGCTGGTT
CAGGTGCCGGTGCTGGTTCAGGTGCTGGTGCTGGTTCAGGTGCTGGTGCTGGTTCAGG
TGCTGGTGCTGGTTCAGGTGCTGGTGCTGGTTCAGGAGCTGGTGCTGGATACGGAGCA

GGGTACGGAGCAGGAGTTGGTGCTGGATACGGAGCAGGAGCTGGCGTTGGATACGGA
GCAGGAGCTGGCGCTGGATACGGAGCAGGAGCTGGAAGCGGAGCTGCCTCTGGTGC
CGGTGCTGGTGCTGGTTCAGGTGCCGGTGCTGGTACAGGTGCTGGGGCTGGTTCAGGA
GCTGGTGCTGGATACGGAGCAGGAGCAGGAAGCGGAGCTGCCTCTGGTGCTGGTGCC
GGTGCTGGAGCTGGTGCAGGAACAGGCTCTTCTGGATTTGGACCATATGTAGCAAATG
GCGGATATAGCGGCTACGAATACGCTTGGTCGTCAGAATCTGACTTTGGAACTGGAAG
CGGAGCTGGTGCTGGTTCAGGTGCTGGTGCTGGTTCAGGTGCTGGTGCTGGCTCAGGT
GCTGGTGCTGGTTCAGGAGCTGGAGCTGGATACGGAGCAGGAGTTGGTGCTGGATAC
GGAGCAGGAGCAGGAAGCGGAGCTGGCTCAGGTGCTGGTGCTGGTTCAGGAGCTGG
AGCTGGTTCAGGTGCTGGTGCTGGTTCAGGAGCTGGTGCTGGTTCAGGAGCTGGAGCT
GGATACGGAGCAGGAGCAGGAAGCGGAACTGGCTCAGGTGCTGGTGCTGGTTCAGGT
GCTGGTGCTGGTTCAGGTGCCGGTGCTGGTTCAGGTGCTGGTGCTGGTTCAGGAGCTG
GTGCTGGTTCAGGTGCTGGTGCTGGTTCAGGAGTTGGTGCTGGATACGGAGTAGGATA
TGGAGCAGGAGCTGGTGCTGGATACGGAGTAGGATATGGAGCAGGAGCTGGTGCTGG
ATACGGAGCAGGAGCAGGAAGCGGAACTGGCTCAGGTGCTGGTGCTGGTTCAGGTGC
TGGTGCTGGTTCAGGTGCCGGTGCTGGTTCAGGTGCTGGTGCTGGTTCAGGAGCTGGT
GCTGGTTCAGGTGCTGGTGCTGGTTCAGGAGCTGGAGCTGGATACGGAGCAGGAGTT
GGTGCTGGATACGGAGTAGGATATGGAGCAGGAGCTGGTGCTGGATACGGAGCAGGA
GCAGGAAGCGGAGCTGGCTCAGGTGCTGGTGCTGGTTCAGGTGCTGGTGCTGGTTCA
GGTGCCGGTGCTGGTTCAGGTGCTGGTGCTGGTTCAGGTGCCGGTTCAGGTGCTGGTG
CTGGTTCAGGAGCTGGTGCTGGTTCAGGTGCTGGTGCTGGTTCAGGTGCTGGTTCAGG
TGCTGGTGCTGGCTCAGGTGCTGGTGCTGGATACGGAGTAGGATATGGAGCAGGAGCT
GGTGCTGGATACGGAGCAGGAGCAGGAAGCGGAGCTGGCTCAGGTGCTGGTGCTGG
GTCAGGTGCCGGTGCTGGTTCAGGTGCTGGTGCTGGTTCAGGTGCCGGTTCAGGTGCT
GGTGCTGGTTCAGGAGCTGGTGCTGGTTCAGGTGCTGGTGCTGGTTCAGGAGCTGGAG
CTGGATACGGAGCAGGAGTTGGTGCTGGATACGGAGTAGGATATGGAGCAGGAGCTG
GTGCTGGATACGGAGCAGGAGCAGGAAGCGGAGCTGGCTCAGGTGCTGGTGCTGGTT
CAGGTGCTGGTGCTGGTTCAGGTGCCGGTGCTGGTTCAGGTGCTGGTGCTGGTTCAGG
AGCTGGTGCTGGTTCAGGTGCCGGTGCTGGTTCAGGTGCTGGTGCTGGTTCAGGTGCC
GGTTCAGGTGCTGGTGCTGGTTCAGGAGCTGGTGCTGGTTCAGGTGCTGGTGCTGGTT
CAGGAGCTGGAGCTGGATACGGAGCAGGAGTTGGTGCTGGATACGGAGTAGGATATG
GAGCAGGAGCTGGTGCTGGATACGGAGCAGGAGCAGGAAGCGGAGCTGCCTCTGGT
GCTGGTGCCGGTGCTGGAGCTGGTGCAGGAACAGGCTCTTCTGGATTTGGACCATATG
TAGCAAATGGCGGATATAGCGGCTACGAATACGCTTGGTCGTCAGAATCTGACTTTGG
AACTGGAAGCGGAGCTGGTGCTGGTTCAGGTGCTGGTGCTGGTTCAGGAGCTGGTGC
TGGATACGGAGCAGGGTACGGAGCAGGAGTTGGTGCTGGATACGGAGCAGGAGCTG
GCGTTGGATACGGAGCAGGAGCTGGCGCTGGATACGGAGCAGGAGCTGGAAGCGGA
GCTGCCTCTGGTGCCGGTGCTGGTGCCGGTGCTGGTGCTGGTTCAGGTGCCGGTGCTG
GTTCAGGTGCTGGTGCTGGTGCTGGTTCAGGAGCTGGTGCTGGATACGGAGCAGGGTA
CGGAATAGGAGTTGGTGCTGGATACGGAGCAGGAGCTGGCGTTGGATACGGAGCAGG
AGCTGGCGCTGGATACGGAGCAGGAGCTGGAAGCGGAGCTGCCTCTGGTGCCGGTGC
TGGTTCAGGTGCTGGTGCTGGTTCAGGTGCTGGTGCTGGTTCAGGTGCTGGTGCTGGTT
CAGGTGCTGGTGCTGGTTCAGGTGCTGGTGCTGGTTCAGGAGCTGGTGCTGGATACGG
AGCAGGGTACGGAGCAGGAGTTGGTGCTGGATACGGAGCAGGAGCTGGCGTTGGATA

CGGAGCAGGAGCTGGCGCTGGATACGGAGCAGGAGCTGGAAGCGGAGCTGCCTCTG
GTGCCGGTGCTGGTGCCGGTGCTGGTGCTGGTGCTGGTTCAGGTGCCGGTGCTGGTTC
AGGTGCTGGTGCTGGTTCAGGTGCTGGTGCTGGTTCAGGAGCTGGTGCTGGTTCAGGT
GCTGGTGCTGGTTCAGGTGCTGGTGCTGGTTCAGGTGCTGGTGCTGGCTCAGGTGCTG
GTGCTGGTTCAGGAGCTGGAGCTGGATACGGAGCAGGAGTTGGTGCTGGATACGGAG
CAGGATATGGAGGAGCTGGTGCTGGATACGGAGCAGGAGCAGGAAGCGGAGCTGCC
TCTGGTGCCGGTGCTGGTTCAGGTGCTGGTGCTGGTTCAGGAGCTGGTGCTGGTTCAG
GTGCTGGTGCTGGTTCAGGTGCTGGGGCTGGTTCAGGTGCTGGTGCTGGATACGGAGC
AGGAGCAGGAAGCGGAGCTGCCTCTGGTGCTGGTGCCGGTGCTGGAGCTGGTGCAGG
AACAGGCTCTTCTGGATTTGGACCATATGTAAATGGCGGATATAGCGGCTACGAATACG
CTTGGTCGTCAGAATCTGACTTTGGAACTGGAAGCGGAGCTGGTGCTGGCTCAGGTGC
TGGTGCTGGTTCAGGAGCTGGAGCTGGATACGGAGCAGGAGTTGGTGCTGGATACGG
AGCAGGATATGGAGCAGGAGCTGGTGCTGGATACGGAGCAGGAGCAGGAAGCGGAG
CTGCCTCTGGTGCCGGTGCTGGTTCAGGTGCTGGTGCTGGTTCAGGTGCCGGTGCTGG
TTCAGGTGCTGGTGCTGGTTCGGGTGCTGGTTCAGGTGCTGGTGCTGGTTCAGGTGCTG
GTGCTGGCTCAGGTGCTGGTGCTGGTTCAGGTGCTGGTGCTGGTTCAGGAGCTGGAGC
TGGATACGGAGCAGGAGTTGGTGTTGGATACGGAGCAGGATATGGAGCAGGAGCTGG
TGCTGGATACGGAGCAGGAGCAGGAAGCGGAGCTGCCTCTGGCGCCGGTGCTGGTTC
AGGTGCTGGTGCTGGTGCTGGTTCAGGTGCCGGTGCTGGTTCAGGTGCTGGTGCTGGT
TCAGGTGCTGGTGCTGGTTCAGGTGCTGGTGCTGGTTCAGGTGCTGGTGCTGGTTCAG
GTGCTGGTTCAGGTGCTGGTGCTGGTTCAGGAGCTGGTGCTGGATACGGAGCAGGGTA
CGGAGCAGGAGTTGGTGCTGGATACGGAGCAGGAGCTGGCGTTGGATACGGAGCAG
GAGCTGGCGCTGGATACGGAGCAGGAGCTGGAAGCGGAGCTGCCTCTGGTGCCGGTG
CTGGTTCAGGTTCTGGTGCTGGTTCAGGTGCCGGTGCTGGTTCAGGTGCTGGTGCTGGT
TCAGGTGCTGGTGCTGGTGCTGGTTCAGGTGCTGGGGCTGGTTCAGGAGCTGGTGCTG
GTTCAGGAGCTGGTGCTGGATACGGAGCAGGGTACGGAGCAGGAGCAGGAAGCGGA
GCTGCCTCTGGTGCTGGTGCCGGTGCTGGAGCTGGTGCAGGAACAGGCTCTTCTGGAT
TTGGACCATATGTAGCAAATGGCGGATATAGCGGCTACGAATACGCTTGGTCGTCAGA
ATCTGACTTTGGAACTGGAAGCGGAGCTGGTGCTGGCTCAGGTGCTGGTGCTGGTTCA
GGAGCTGGAGCTGGATACGGAGCAGGAGTTGGTGCTGGTTACGGAGCAGGATATGGA
GCAGGAGCTGGTGCTGGATACGGAGCAGGAGCAGGAAGCGGAGCTGCCTCAGGTGC
CGGTGCTGGTTCAGGTGCTGGTGCTGGTTCAGGTGCCGGTGCTGGTTCAGGTGCTGGT
GCTGGTTCAGGAGCTGGTGCTGGTTCAGGTGCTGGTGCTGGTTCAGGTGCTGGTGCTG
GTTCAGGTGCTGGTGCTGGTTCAGGAGCTGGTGCTGGATACGGAGCAGGGTACGGAG
CAGGAGTTGGTGCTGGATACGGAGCAGGAGCTGGCGTTGGATACGGAGCAGGAGCTG
GCGCTGGATACGGAGCAGGAGCTGGAAGCGGAGCTGCCTCTGGTGCCGGTGCTGGTT
CAGGTTCTGGTGCTGGTGCTGGTTCAGGTTCTGGTGCTGGTTCAGGTGCTGGTGCTGGT
TCAGGTGCTGGTGCTGGTTCAGGTGCTGGTGCTGGTTCAGGAGCTGGTGCTGGTTCAG
GTGCTGGTGCTGGTTCAGGTGCTGGTGCTGGTTCAGGTGCTGGTGCTGGATACGGAGC
AGGGTACGGAATAGGAGTTGGTGCTGGATACGGAGCAGGAGCTGGCGTTGGATACGG
AGCAGGAGCTGGCGCTGGATACGGAGCAGGAGCTGGAAGCGGAGCTGCCTCTGGTG
CCGGTGCTGGTTCAGGTGCTGGTGCTGGTTCAGGAGCTGGTGCTGGTTCAGGTGCTGG
TGCTGGTTCAGGTGCTGGTGCTGGTTCAGGTGCTGGTGCTGGTTCAGGAGCTGGTGCT
GGTTCAGGTGCTGGTGCTGGTTCAGGTGCTGGTGCTGGTTCAGGAGCTGGTGCTGGAT

ACGGAGCAGGAGCTGGCGTTGGATACGGAGCAGGAGCTGGAAGCGGAGCTGCCTCT
GGTGCTGGTGCTGGTTCAGGTGCTGGTGCTGGTTCAGGTGCTGGTGCTGGTTCAGGTG
CTGGTGCTGGTTCAGGTGCTGGTGCTGGTTCAGGTGCTGGTGCTGGTTCAGGTGCTGGT
GCTGGTTCAGGTGCTGGTTCAGGTGCTGGTGCTGGCTCAGGTGCTGGTGCTGGATACG
GAGCAGGGTACGGAGCAGGAGTTGGTGCTGGATACGGAGCAGGAGCTGGCGTTGGAT
ACGGAGCAGGATATGGAGTAGGAGCTGGTGCTGGATACGGAGCAGGAGCAGGAAGC
GGAGCTGCCTCTGGTGCTGGTGCTGGTTCAGGTGCTGGTGCTGGTTCAGGTGCTGGTG
CTGGTTCAGGTGCTGGTGCTGGTTCAGGTGCTGGTGCTGGTTCAGGTGCTGGTTCAGG
AGCTGGTGCTGGATACGGAGCAGGAGCTGGCGTTGGATACGGAGCAGGAGCTGGCGC
TGGATACGGAGCAGGAGCTGGAAGCGGAGCTGCCTCTGGTGCTGGTGCTGGTGCCGG
TGCTGGTTCAGGTGCTGGTGCTGGTTCAGGTGCTGGTGCTGGTTCAGGTGCTGGTTCA
GGTGCTGGTGCTGGTTCAGGAGCTGGTGCTGGATACGGAGCAGGAGCTGGAAGCGGA
GCTGCCTCTGGTGCCGGTGCTGGTTCAGGTGCTGGTGCTGGTGCTGGTGCCGGTGCTG
GTGCTGGTTCAGGAGCTGGTGCTGGTTCAGGAGCTGGTGCTGGATACGGAGCAGGAG
CAGGAAGTGGAGCTGCCTCTGGTGCTGGTGCTGGAGCTGGTGCAGGAACAGGCTCTT
CTGGATTTGGACCATATGTAGCAAATGGCGGATATAGCAGACGTGAAGGCTACGAATA
CGCTTGGTCGTCAAAATCTGACTTTGAAACTGGAAGCGGTGCTGCCTCTGGTGCTGGT
GCTGGTGCTGGTTCAGGTGCTGGTGCTGGTTCAGGTGCCGGTGCTGGTTCAGGTGCTG
GTGCTGGTTCAGGTGCCGGTGCTGGTGGTAGCGTCAGTTACGGAGCTGGCAGGGGATA
CGGACAAGGTGCAGGAAGTGCAGCTTCCTCTGTGTCATCTGCTTCATCTCGCAGTTAC
GACTATTCTCGTCGTAACGTCCGCAAAAACTGTGGAATTCCTAGAAGACAACTAGTTG
TTAAATTCAGAGCACTGCCTTGTGTGAATTGCTAA

The consensus sequence contains two exons and one intron. The black and gray letters are exons and introns, respectively.

Supplementary Table S1 | Primers used in this study

Primer name	Sequence (5' to 3')
lw-Cpf1-up-F1	TTTGAATAAGTGCTTAATTGCAAG
lw-Cpf1-up-R1	CGTAACTACAACCTACAGTGGAG
lw-Cpf1-up-F2	GTCGTCTAATGCAAAGATTATTGG
lw-Cpf1-up-R2	CTGGAGAATGCCTTGGATG
lw-Cpf1-down-F1	AACTGTGGAATTCCTAGAAGACAA
lw-Cpf1-down-R1	ATATCTACACCCACAACTTTTAGCG
lw-Cpf1-down-F2	CCCGGTCTCAGGAGACATTAG
lw-Cpf1-down-R2	CGTCGAAAACACAGGACATTG

**Supplementary Table S2 | ** Called sites methylated on the reference gene

chromosome	start	end	num_motifs_in_group	called_sites	called_sites_methylated	methylated_frequency	group_sequence
Bomo_Chr25	10354653	10354653	1	32	4	0.125	AAATCCGAAAT
Bomo_Chr25	10354715	10354715	1	38	2	0.053	ATTAACGAAAA
Bomo_Chr25	10354744	10354744	1	44	1	0.023	CAGGACGAAGT
Bomo_Chr25	10354849	10354879	6	480	0	0	TTTTGCGGACGTTACGACGAGAATAGTCGTAACTGCGAGAT
Bomo_Chr25	10354928	10354928	1	41	2	0.049	TTGTCCGTATC
Bomo_Chr25	10354946	10354955	2	98	4	0.041	AGCTCCGTAACTGACGCTAC
Bomo_Chr25	10354970	10354970	1	52	4	0.077	AGCACCGGCAC
Bomo_Chr25	10355006	10355006	1	50	3	0.06	AGCACCGGCAC
Bomo_Chr25	10355066	10355066	1	49	1	0.02	AGCACCGCTTC
Bomo_Chr25	10355094	10355108	3	219	0	0	TTTGACGACCAAGCGTATTCGTAGC
Bomo_Chr25	10355119	10355119	1	29	1	0.034	CTTCACGTCTG
Bomo_Chr25	10355132	10355132	1	22	1	0.045	ATATCCGCCAT
Bomo_Chr25	10355228	10355228	1	32	2	0.062	TGCTCCGTATC
Bomo_Chr25	10355282	10355282	1	50	0	0	AGCACCGGCAC
Bomo_Chr25	10355318	10355318	1	47	0	0	AGCACCGGCAC
Bomo_Chr25	10355336	10355336	1	40	1	0.025	AGCTCCGCTTC
Bomo_Chr25	10355354	10355354	1	35	0	0	TGCTCCGTATC
Bomo_Chr25	10355450	10355450	1	49	0	0	AGCACCGGCAC
Bomo_Chr25	10355480	10355480	1	38	2	0.053	AGCTCCGCTTC
Bomo_Chr25	10355498	10355507	2	124	4	0.032	TGCTCCGTATCCAGCGCCAG
Bomo_Chr25	10355522	10355531	2	148	0	0	TGCTCCGTATCCAACGCCAG
Bomo_Chr25	10355546	10355546	1	36	0	0	TGCTCCGTATC
Bomo_Chr25	10355678	10355678	1	48	4	0.083	AGCTCCGCTTC

Bomo_Chr25	10355696	10355696	1	39	0	0	TGCTCCGTATC
Bomo_Chr25	10355732	10355741	2	128	0	0	TGCTCCGTATCCAACGCCAG
Bomo_Chr25	10355756	10355756	1	40	0	0	TGCTCCGTATC
Bomo_Chr25	10355780	10355780	1	30	0	0	TGCTCCGTACC
Bomo_Chr25	10355792	10355792	1	33	0	0	TGCTCCGTATC
Bomo_Chr25	10355978	10355978	1	39	4	0.103	AGCTCCGCTTC
Bomo_Chr25	10355996	10356005	2	138	2	0.014	TGCTCCGTATCCAACGCCAG
Bomo_Chr25	10356020	10356020	1	41	0	0	TGCTCCGTATC
Bomo_Chr25	10356194	10356194	1	56	0	0	AGCACCGGCAC
Bomo_Chr25	10356212	10356212	1	36	1	0.028	AGCTCCGCTTC
Bomo_Chr25	10356230	10356239	2	126	4	0.032	TGCTCCGTATCCAGCGCCAG
Bomo_Chr25	10356254	10356263	2	132	0	0	TGCTCCGTATCCAACGCCAG
Bomo_Chr25	10356278	10356278	1	39	2	0.051	TGCTCCGTATC
Bomo_Chr25	10356302	10356302	1	34	1	0.029	TATTCCGTACC
Bomo_Chr25	10356314	10356314	1	38	1	0.026	TGCTCCGTATC
Bomo_Chr25	10356494	10356494	1	49	1	0.02	AGCACCGGCAC
Bomo_Chr25	10356512	10356512	1	45	4	0.089	AGCTCCGCTTC
Bomo_Chr25	10356530	10356539	2	138	14	0.101	TGCTCCGTATCCAGCGCCAG
Bomo_Chr25	10356554	10356563	2	142	0	0	TGCTCCGTATCCAACGCCAG
Bomo_Chr25	10356578	10356578	1	44	1	0.023	TGCTCCGTATC
Bomo_Chr25	10356602	10356602	1	39	3	0.077	TGCTCCGTACC
Bomo_Chr25	10356614	10356614	1	39	2	0.051	TGCTCCGTATC
Bomo_Chr25	10356734	10356734	1	44	0	0	AGCACCGGCAC
Bomo_Chr25	10356770	10356770	1	54	0	0	AGCACCGGCAC
Bomo_Chr25	10356788	10356788	1	46	5	0.109	AGCTCCGCTTC
Bomo_Chr25	10356806	10356806	1	45	1	0.022	TGCTCCGTATC

Bomo_Chr25	10356842	10356842	1	42	0	0	TGCTCCGTAAC
Bomo_Chr25	10356866	10356866	1	42	2	0.048	TGCTCCGTATC
Bomo_Chr25	10356920	10356920	1	32	2	0.062	AGCTCCGCTTC
Bomo_Chr25	10356948	10356977	5	370	10	0.027	TCTGACGACCAAGCGTATTCGTAGCCGCTATATCCGCCAT
Bomo_Chr25	10357037	10357037	1	53	0	0	AGCACCGGCAC
Bomo_Chr25	10357061	10357061	1	46	2	0.043	AGCTCCGCTTC
Bomo_Chr25	10357079	10357079	1	43	0	0	TGCTCCGTACC
Bomo_Chr25	10357091	10357091	1	43	1	0.023	TGCTCCGTATC
Bomo_Chr25	10357199	10357199	1	42	2	0.048	AGCACCGGCAC
Bomo_Chr25	10357235	10357235	1	59	0	0	AGCACCGGCAC
Bomo_Chr25	10357253	10357253	1	45	5	0.111	AGCTCCGCTTC
Bomo_Chr25	10357271	10357280	2	130	2	0.015	TGCTCCGTATCCAGCGCCAG
Bomo_Chr25	10357295	10357304	2	144	0	0	TGCTCCGTATCCAACGCCAG
Bomo_Chr25	10357319	10357319	1	42	1	0.024	TGCTCCGTATC
Bomo_Chr25	10357343	10357343	1	40	0	0	TGCTCCGTACC
Bomo_Chr25	10357355	10357355	1	37	0	0	TGCTCCGTATC
Bomo_Chr25	10357487	10357487	1	39	2	0.051	AGCACCGGCAC
Bomo_Chr25	10357529	10357532	2	140	0	0	AGCACCGGCGCCAG
Bomo_Chr25	10357547	10357547	1	37	3	0.081	AGCTCCGCTTC
Bomo_Chr25	10357565	10357565	1	45	0	0	TGCTCCGTATC
Bomo_Chr25	10357601	10357601	1	32	1	0.031	TGCTCCGTATC
Bomo_Chr25	10357625	10357625	1	44	0	0	TGCTCCGTATC
Bomo_Chr25	10357728	10357728	1	28	1	0.036	GCACCCGAACC
Bomo_Chr25	10357757	10357757	1	48	1	0.021	AGCACCGGCAC
Bomo_Chr25	10357793	10357793	1	46	3	0.065	AGCACCGGCAC
Bomo_Chr25	10357811	10357811	1	41	3	0.073	AGCTCCGCTTC

Bomo_Chr25	10357829	10357829	1	44	0	0	TGCTCCGTATC
Bomo_Chr25	10357865	10357865	1	42	1	0.024	TGCTCCGTATC
Bomo_Chr25	10357889	10357889	1	38	1	0.026	TGCTCCGTATC
Bomo_Chr25	10357943	10357943	1	36	3	0.083	AGCTCCGCTTC
Bomo_Chr25	10357971	10358000	5	365	5	0.014	TCTGACGACCAAGCGTATTCGTAGCCGCTATATCCGCCAT
Bomo_Chr25	10358057	10358057	1	47	1	0.021	AGCACCGGCAC
Bomo_Chr25	10358081	10358081	1	39	0	0	AGCTCCGCTTC
Bomo_Chr25	10358099	10358099	1	53	2	0.038	TGCTCCGTATC
Bomo_Chr25	10358201	10358201	1	51	1	0.02	AGCACCGGCAC
Bomo_Chr25	10358219	10358219	1	40	2	0.05	AGCTCCGCTTC
Bomo_Chr25	10358237	10358237	1	38	1	0.026	TGCTCCGTATC
Bomo_Chr25	10358270	10358270	1	41	3	0.073	TGCTCCGTATC
Bomo_Chr25	10358294	10358294	1	40	0	0	TGCTCCGTATC
Bomo_Chr25	10358450	10358450	1	39	1	0.026	AGCACCGGCAC
Bomo_Chr25	10358480	10358480	1	39	0	0	AGCACCGGCAC
Bomo_Chr25	10358492	10358492	1	52	0	0	AGCACCGGCAC
Bomo_Chr25	10358510	10358510	1	43	3	0.07	AGCTCCGCTTC
Bomo_Chr25	10358528	10358537	2	126	2	0.016	TGCTCCGTATCCAGCGCCAG
Bomo_Chr25	10358552	10358561	2	150	0	0	TGCTCCGTATCCAACGCCAG
Bomo_Chr25	10358576	10358576	1	42	0	0	TGCTCCGTATC
Bomo_Chr25	10358600	10358600	1	46	3	0.065	TGCTCCGTACC
Bomo_Chr25	10358612	10358612	1	44	1	0.023	TGCTCCGTATC
Bomo_Chr25	10358732	10358732	1	52	2	0.038	AGCACCGGCAC
Bomo_Chr25	10358750	10358750	1	39	1	0.026	AGCTCCGCTTC
Bomo_Chr25	10358768	10358777	2	124	2	0.016	TGCTCCGTATCCAGCGCCAG
Bomo_Chr25	10358792	10358801	2	146	0	0	TGCTCCGTATCCAACGCCAG

Bomo_Chr25	10358816	10358816	1	42	0	0	TGCTCCGTATC
Bomo_Chr25	10358840	10358840	1	39	0	0	TATTCCGTACC
Bomo_Chr25	10358852	10358852	1	44	0	0	TGCTCCGTATC
Bomo_Chr25	10358906	10358906	1	43	0	0	AGCACCGGCAC
Bomo_Chr25	10358930	10358930	1	44	1	0.023	AGCACCGGCAC
Bomo_Chr25	10358942	10358942	1	55	1	0.018	AGCACCGGCAC
Bomo_Chr25	10358960	10358960	1	41	1	0.024	AGCTCCGCTTC
Bomo_Chr25	10358978	10358987	2	130	2	0.015	TGCTCCGTATCCAGCGCCAG
Bomo_Chr25	10359002	10359011	2	146	0	0	TGCTCCGTATCCAACGCCAG
Bomo_Chr25	10359026	10359026	1	45	1	0.022	TGCTCCGTATC
Bomo_Chr25	10359050	10359050	1	38	0	0	TGCTCCGTACC
Bomo_Chr25	10359062	10359062	1	39	0	0	TGCTCCGTATC
Bomo_Chr25	10359116	10359116	1	42	3	0.071	AGCTCCGCTTC
Bomo_Chr25	10359144	10359173	5	345	0	0	TCTGACGACCAAGCGTATTCGTAGCCGCTATATCCGCCAT
Bomo_Chr25	10359233	10359233	1	47	0	0	AGCACCGGCAC
Bomo_Chr25	10359257	10359257	1	42	4	0.095	AGCTCCGCTTC
Bomo_Chr25	10359275	10359275	1	42	2	0.048	TGCTCCGTATC
Bomo_Chr25	10359311	10359311	1	43	0	0	TACTCCGTATC
Bomo_Chr25	10359335	10359335	1	44	1	0.023	TGCTCCGTATC
Bomo_Chr25	10359413	10359413	1	40	3	0.075	TGAACCGGCAC
Bomo_Chr25	10359449	10359449	1	42	2	0.048	AGCACCGGCAC
Bomo_Chr25	10359503	10359503	1	50	0	0	AGCACCGGCAC
Bomo_Chr25	10359557	10359557	1	44	4	0.091	AGCTCCGCTTC
Bomo_Chr25	10359575	10359575	1	40	1	0.025	TGCTCCGTATC
Bomo_Chr25	10359611	10359611	1	44	1	0.023	TACTCCGTATC
Bomo_Chr25	10359635	10359635	1	41	0	0	TGCTCCGTATC

Bomo_Chr25	10359713	10359713	1	40	0	0	TGAACCGGCAC
Bomo_Chr25	10359749	10359749	1	34	2	0.059	AGCACCGGCAC
Bomo_Chr25	10359785	10359785	1	43	1	0.023	AGCTCCGCTTC
Bomo_Chr25	10359803	10359803	1	46	3	0.065	TGCTCCGTATC
Bomo_Chr25	10359839	10359839	1	43	1	0.023	TACTCCGTATC
Bomo_Chr25	10359947	10359947	1	39	0	0	TGAACCGGCAC
Bomo_Chr25	10359983	10359983	1	37	0	0	AGCACCGGCAC
Bomo_Chr25	10360037	10360037	1	40	2	0.05	AGCTCCGCTTC
Bomo_Chr25	10360055	10360055	1	47	4	0.085	TGCTCCGTATC
Bomo_Chr25	10360091	10360091	1	45	4	0.089	TACTCCGTATC
Bomo_Chr25	10360115	10360115	1	41	1	0.024	TGCTCCGTATC
Bomo_Chr25	10360199	10360199	1	40	1	0.025	AGCACCGGCAC
Bomo_Chr25	10360253	10360253	1	31	5	0.161	AGTTCCGCTTC
Bomo_Chr25	10360271	10360271	1	44	2	0.045	TGCTCCGTATC
Bomo_Chr25	10360307	10360307	1	42	1	0.024	TACTCCGTATC
Bomo_Chr25	10360343	10360343	1	43	1	0.023	TACTCCGTATC
Bomo_Chr25	10360427	10360427	1	50	1	0.02	AGCACCGGCAC
Bomo_Chr25	10360481	10360481	1	34	7	0.206	AGTTCCGCTTC
Bomo_Chr25	10360499	10360499	1	39	1	0.026	TGCTCCGTATC
Bomo_Chr25	10360601	10360601	1	38	4	0.105	AGCTCCGCTTC
Bomo_Chr25	10360619	10360619	1	48	1	0.021	TGCTCCGTATC
Bomo_Chr25	10360643	10360643	1	48	1	0.021	TGCTCCGTATC
Bomo_Chr25	10360733	10360733	1	36	5	0.139	AGCTCCGCTTC
Bomo_Chr25	10360761	10360790	5	350	10	0.029	TCTGACGACCAAGCGTATTCGTAGCCGCTATATCCGCCAT
Bomo_Chr25	10360850	10360850	1	49	1	0.02	AGCACCGGCAC
Bomo_Chr25	10360874	10360874	1	38	2	0.053	AGCTCCGCTTC

Bomo_Chr25	10360892	10360892	1	47	0	0	TGCTCCGTATC
Bomo_Chr25	10360940	10360940	1	44	0	0	AGCACCGGCAC
Bomo_Chr25	10360964	10360964	1	53	0	0	AGCACCGGCAC
Bomo_Chr25	10360982	10360982	1	43	1	0.023	AGCTCCGCTTC
Bomo_Chr25	10361000	10361009	2	136	2	0.015	TGCTCCGTATCCAGCGCCAG
Bomo_Chr25	10361024	10361033	2	144	0	0	TGCTCCGTATCCAACGCCAG
Bomo_Chr25	10361048	10361048	1	47	1	0.021	TGCTCCGTATC
Bomo_Chr25	10361072	10361072	1	42	1	0.024	TGCTCCGTACC
Bomo_Chr25	10361084	10361084	1	38	0	0	TGCTCCGTATC
Bomo_Chr25	10361186	10361186	1	45	0	0	AGCACCGGCAC
Bomo_Chr25	10361228	10361228	1	52	0	0	AGCACCGGCAC
Bomo_Chr25	10361246	10361246	1	43	3	0.07	AGCTCCGCTTC
Bomo_Chr25	10361264	10361264	1	46	1	0.022	TGCTCCGTATC
Bomo_Chr25	10361300	10361300	1	46	0	0	TACTCCGTATC
Bomo_Chr25	10361324	10361324	1	43	1	0.023	TGCTCCGTATC
Bomo_Chr25	10361402	10361402	1	43	0	0	TGAACCGGCAC
Bomo_Chr25	10361438	10361438	1	47	2	0.043	AGCACCGGCAC
Bomo_Chr25	10361486	10361486	1	48	0	0	TGAACCGGCAC
Bomo_Chr25	10361522	10361522	1	45	0	0	AGCACCGGCAC
Bomo_Chr25	10361576	10361576	1	44	3	0.068	AGCACCGGCAC
Bomo_Chr25	10361630	10361630	1	43	2	0.047	AGCTCCGCTTC
Bomo_Chr25	10361648	10361648	1	46	0	0	TGCTCCGTATC
Bomo_Chr25	10361684	10361684	1	49	0	0	TACTCCGTATC
Bomo_Chr25	10361708	10361708	1	47	3	0.064	TGCTCCGTATC
Bomo_Chr25	10361786	10361786	1	41	1	0.024	TGAACCGGCAC
Bomo_Chr25	10361822	10361822	1	39	4	0.103	AGCACCGGCAC

Bomo_Chr25	10361858	10361858	1	48	5	0.104	AGCTCCGCTTC
Bomo_Chr25	10361876	10361876	1	42	0	0	TGCTCCGTATC
Bomo_Chr25	10361912	10361912	1	41	1	0.024	TACTCCGTATC
Bomo_Chr25	10362020	10362020	1	42	1	0.024	TGAACCGGCAC
Bomo_Chr25	10362056	10362056	1	49	2	0.041	AGCACCGGCAC
Bomo_Chr25	10362110	10362110	1	43	2	0.047	AGCTCCGCTTC
Bomo_Chr25	10362128	10362128	1	51	2	0.039	TGCTCCGTATC
Bomo_Chr25	10362164	10362164	1	51	0	0	TACTCCGTATC
Bomo_Chr25	10362188	10362188	1	45	1	0.022	TGCTCCGTATC
Bomo_Chr25	10362272	10362272	1	43	1	0.023	AGCACCGGCAC
Bomo_Chr25	10362308	10362308	1	60	2	0.033	AGCACCGGCAC
Bomo_Chr25	10362326	10362326	1	41	0	0	AGCTCCGCTTC
Bomo_Chr25	10362344	10362344	1	51	0	0	TGCTCCGTATC
Bomo_Chr25	10362392	10362392	1	45	2	0.044	AGCACCGGCAC
Bomo_Chr25	10362428	10362428	1	52	1	0.019	AGCACCGGCAC
Bomo_Chr25	10362482	10362482	1	46	1	0.022	AGCTCCGCTTC
Bomo_Chr25	10362500	10362500	1	56	2	0.036	TGCTCCGTATC
Bomo_Chr25	10362536	10362536	1	51	2	0.039	TGCTCCGTATC
Bomo_Chr25	10362650	10362650	1	38	5	0.132	AGCTCCGCTTC
Bomo_Chr25	10362678	10362707	5	395	0	0	TCTGACGACCAAGCGTATTCGTAGCCGCTATATCCGCCAT
Bomo_Chr25	10362767	10362767	1	50	0	0	AGCACCGGCAC
Bomo_Chr25	10362791	10362791	1	48	0	0	AGCTCCGCTTC
Bomo_Chr25	10362809	10362809	1	58	1	0.017	TGCTCCGTATC
Bomo_Chr25	10362839	10362839	1	44	1	0.023	AGCACCGGCAC
Bomo_Chr25	10362923	10362923	1	52	1	0.019	AGCACCGGCAC
Bomo_Chr25	10362965	10362965	1	64	0	0	AGCACCGGCAC

Bomo_Chr25	10362983	10362983	1	39	2	0.051	AGCTCCGCTTC
Bomo_Chr25	10363001	10363001	1	51	0	0	AGCTCCGTATC
Bomo_Chr25	10363037	10363037	1	53	1	0.019	TGCTCCGTATC
Bomo_Chr25	10363061	10363061	1	56	0	0	TGCTCCGTATC
Bomo_Chr25	10363127	10363127	1	48	2	0.042	AGCACCGGCAC
Bomo_Chr25	10363163	10363163	1	58	0	0	AGCACCGGCAC
Bomo_Chr25	10363181	10363181	1	50	1	0.02	AGCTCCGCTTC
Bomo_Chr25	10363199	10363199	1	53	0	0	TGCTCCGTATC
Bomo_Chr25	10363223	10363223	1	45	0	0	TGCTCCGTACC
Bomo_Chr25	10363235	10363235	1	54	0	0	TGCTCCGTATC
Bomo_Chr25	10363319	10363319	1	52	0	0	AGCACCGGCAC
Bomo_Chr25	10363349	10363349	1	70	0	0	AGCACCGGCAC
Bomo_Chr25	10363367	10363367	1	50	4	0.08	AGCTCCGCTTC
Bomo_Chr25	10363385	10363385	1	62	0	0	TGCTCCGTATC
Bomo_Chr25	10363409	10363409	1	61	1	0.016	TGCTCCGTATC
Bomo_Chr25	10363457	10363457	1	42	3	0.071	AGTTCCGCTTC
Bomo_Chr25	10363475	10363475	1	61	1	0.016	TGCTCCGTATC
Bomo_Chr25	10363511	10363511	1	55	1	0.018	TGCTCCGTATC
Bomo_Chr25	10363535	10363535	1	60	0	0	TGCTCCGTATC
Bomo_Chr25	10363574	10363574	1	59	0	0	ACCTGCGCCAG
Bomo_Chr25	10363607	10363607	1	51	3	0.059	AGCTCCGCTTC
Bomo_Chr25	10363635	10363664	5	435	5	0.011	TCTGACGACCAAGCGTATTCGTAGCCGCTATATCCGCCAT
Bomo_Chr25	10363724	10363724	1	61	0	0	AGCACCGGCAC
Bomo_Chr25	10363748	10363748	1	50	4	0.08	AGCTCCGCTTC
Bomo_Chr25	10363766	10363766	1	66	2	0.03	TGCTCCGTATC
Bomo_Chr25	10363862	10363862	1	52	1	0.019	AGCACCGCTTC

Bomo_Chr25	10363880	10363880	1	61	2	0.033	TGCTCCGTATC
Bomo_Chr25	10363916	10363916	1	61	1	0.016	TGCTCCGTATC
Bomo_Chr25	10363940	10363940	1	59	0	0	TGCTCCGTATC
Bomo_Chr25	10364006	10364006	1	50	2	0.04	AGCACCGGCAC
Bomo_Chr25	10364060	10364060	1	60	2	0.033	AGAACCGGCAC
Bomo_Chr25	10364096	10364096	1	53	0	0	AGCACCGGCAC
Bomo_Chr25	10364186	10364186	1	54	5	0.093	AGCTCCGCTTC
Bomo_Chr25	10364204	10364204	1	52	1	0.019	TGCTCCGTATC
Bomo_Chr25	10364240	10364240	1	54	0	0	TGCTCCGTATC
Bomo_Chr25	10364294	10364294	1	55	1	0.018	AGCACCGGCAC
Bomo_Chr25	10364312	10364312	1	49	0	0	AGCTCCGCTTC
Bomo_Chr25	10364330	10364330	1	57	0	0	TGCTCCGTATC
Bomo_Chr25	10364366	10364366	1	61	0	0	TGCTCCGTATC
Bomo_Chr25	10364390	10364390	1	59	1	0.017	TGCTCCGTATC
Bomo_Chr25	10364456	10364456	1	57	1	0.018	AGAACCGGCAC
Bomo_Chr25	10364492	10364492	1	49	1	0.02	AGCACCGGCAC
Bomo_Chr25	10364564	10364564	1	51	0	0	AGCTCCGCTTC
Bomo_Chr25	10364582	10364582	1	62	0	0	TGCTCCGTATC
Bomo_Chr25	10364636	10364636	1	57	1	0.018	AGCACCGGCAC
Bomo_Chr25	10364762	10364762	1	52	0	0	AGCACCGGCAC
Bomo_Chr25	10364798	10364798	1	58	1	0.017	AGCACCGGCAC
Bomo_Chr25	10364816	10364816	1	55	2	0.036	AGCTCCGCTTC
Bomo_Chr25	10364834	10364834	1	54	1	0.019	AGCTCCGTATC
Bomo_Chr25	10364870	10364870	1	60	0	0	TGCTCCGTATC
Bomo_Chr25	10364894	10364894	1	61	0	0	TGCTCCGTATC
Bomo_Chr25	10365020	10365020	1	46	2	0.043	AGCTCCGCTTC

Bomo_Chr25	10365048	10365077	5	445	10	0.022	TCTGACGACCAAGCGTATTCGTAGCCGCTATATCCGCCAT
Bomo_Chr25	10365137	10365137	1	69	1	0.014	AGCACCGGCAC
Bomo_Chr25	10365161	10365161	1	57	3	0.053	AGCTCCGCTTC
Bomo_Chr25	10365179	10365179	1	68	1	0.015	TGCTCCGTATC
Bomo_Chr25	10365215	10365215	1	63	1	0.016	TACTCCGTATC
Bomo_Chr25	10365239	10365239	1	66	0	0	TGCTCCGTATC
Bomo_Chr25	10365305	10365305	1	49	2	0.041	AGAACCGGCAC
Bomo_Chr25	10365359	10365359	1	49	2	0.041	AGCACCGGCAC
Bomo_Chr25	10365395	10365395	1	63	0	0	AGCACCGGCAC
Bomo_Chr25	10365449	10365449	1	55	2	0.036	AGCTCCGCTTC
Bomo_Chr25	10365467	10365467	1	63	0	0	TGCTCCGTATC
Bomo_Chr25	10365503	10365503	1	62	1	0.016	TGCTCCGTATC
Bomo_Chr25	10365593	10365593	1	51	3	0.059	AGCTCCGCTTC
Bomo_Chr25	10365621	10365650	5	455	5	0.011	TCTGACGACCAAGCGTATTCGTAGCCGCTATATCCGCCAT
Bomo_Chr25	10365710	10365710	1	67	2	0.03	AGCACCGGCAC
Bomo_Chr25	10365734	10365734	1	52	1	0.019	AGCTCCGCTTC
Bomo_Chr25	10365752	10365752	1	71	1	0.014	TGCTCCGTATC
Bomo_Chr25	10365788	10365788	1	60	1	0.017	TGCTCCGTATC
Bomo_Chr25	10365812	10365812	1	68	0	0	TGCTCCGTATC
Bomo_Chr25	10365896	10365896	1	55	1	0.018	AGCACCGGCAC
Bomo_Chr25	10365968	10365968	1	58	1	0.017	AGCTCCGCTTC
Bomo_Chr25	10365986	10365986	1	68	0	0	TGCTCCGTATC
Bomo_Chr25	10366022	10366022	1	67	0	0	TGCTCCGTATC
Bomo_Chr25	10366052	10366052	1	53	1	0.019	AGCACCGGCAC
Bomo_Chr25	10366106	10366106	1	61	1	0.016	AACACCGGCAC
Bomo_Chr25	10366178	10366178	1	60	1	0.017	AGCTCCGCTTC

Bomo_Chr25	10366196	10366196	1	65	0	0	TGCTCCGTATC
Bomo_Chr25	10366232	10366232	1	61	0	0	TGCTCCGTATC
Bomo_Chr25	10366256	10366256	1	65	0	0	TGCTCCGTATC
Bomo_Chr25	10366340	10366340	1	60	0	0	AGCACCGGCAC
Bomo_Chr25	10366412	10366412	1	61	3	0.049	AGCTCCGCTTC
Bomo_Chr25	10366430	10366430	1	66	1	0.015	TGCTCCGTATC
Bomo_Chr25	10366466	10366466	1	70	0	0	TGCTCCGTATC
Bomo_Chr25	10366496	10366496	1	64	1	0.016	AGCACCGGCAC
Bomo_Chr25	10366586	10366586	1	57	0	0	AGCACCGGCAC
Bomo_Chr25	10366640	10366640	1	62	4	0.065	AGCTCCGCTTC
Bomo_Chr25	10366658	10366658	1	69	2	0.029	TGCTCCGTATC
Bomo_Chr25	10366694	10366694	1	64	0	0	TGCTCCGTATC
Bomo_Chr25	10366718	10366718	1	70	0	0	TGCTCCGTATC
Bomo_Chr25	10366802	10366802	1	55	2	0.036	AGCTCCGCTTC
Bomo_Chr25	10366830	10366859	5	485	0	0	TCTGACGACCAAGCGTATTCGTAGCCGCTATATCCGCCAT
Bomo_Chr25	10366919	10366919	1	62	1	0.016	AGCACCGGCAC
Bomo_Chr25	10366943	10366943	1	69	1	0.014	AGCTCCGCTTC
Bomo_Chr25	10366961	10366961	1	69	1	0.014	TGCTCCGTATC
Bomo_Chr25	10366997	10366997	1	69	0	0	TGCTCCGTATC
Bomo_Chr25	10367099	10367099	1	69	0	0	AGCACCGGCAC
Bomo_Chr25	10367135	10367135	1	78	0	0	AGCACCGGCAC
Bomo_Chr25	10367153	10367153	1	60	3	0.05	AGCTCCGCTTC
Bomo_Chr25	10367171	10367171	1	71	0	0	TGCTCCGTATC
Bomo_Chr25	10367207	10367207	1	69	0	0	TGCTCCGTATC
Bomo_Chr25	10367231	10367231	1	68	1	0.015	TGCTCCGTATC
Bomo_Chr25	10367357	10367357	1	60	1	0.017	AGCACCGGCAC

Bomo_Chr25	10367375	10367375	1	58	0	0	AGCACCGGCAC
Bomo_Chr25	10367405	10367405	1	56	1	0.018	AGCTCCGCTTC
Bomo_Chr25	10367423	10367423	1	66	0	0	TGCTCCGTATC
Bomo_Chr25	10367459	10367459	1	68	2	0.029	TGCTCCGTATC
Bomo_Chr25	10367483	10367483	1	68	1	0.015	TGCTCCGTATC
Bomo_Chr25	10367657	10367657	1	55	1	0.018	AGCACCGGCAC
Bomo_Chr25	10367693	10367693	1	65	0	0	AGCACCGGCAC
Bomo_Chr25	10367711	10367711	1	62	0	0	AGCTCCGCTTC
Bomo_Chr25	10367729	10367729	1	74	0	0	TGCTCCGTATC
Bomo_Chr25	10367765	10367765	1	72	0	0	TGCTCCGTATC
Bomo_Chr25	10367789	10367789	1	74	3	0.041	TGCTCCGTATC
Bomo_Chr25	10367927	10367927	1	56	2	0.036	AGCACCGGCAC
Bomo_Chr25	10367963	10367963	1	55	0	0	AGCTCCGCTTC
Bomo_Chr25	10367981	10367981	1	64	2	0.031	TGCTCCGTATC
Bomo_Chr25	10368017	10368017	1	68	0	0	TGCTCCGTATC
Bomo_Chr25	10368041	10368041	1	67	1	0.015	TGCTCCGTATC
Bomo_Chr25	10368065	10368065	1	70	0	0	TGCTCCGTATC
Bomo_Chr25	10368167	10368167	1	53	1	0.019	AGCACCGGCAC
Bomo_Chr25	10368203	10368203	1	56	0	0	AGCACCGGCAC
Bomo_Chr25	10368239	10368239	1	64	1	0.016	AGCACCGGCAC
Bomo_Chr25	10368275	10368275	1	60	2	0.033	AGCACCGGCAC
Bomo_Chr25	10368311	10368311	1	75	0	0	AGCACCGGCAC
Bomo_Chr25	10368329	10368329	1	53	2	0.038	AGCTCCGCTTC
Bomo_Chr25	10368347	10368347	1	76	0	0	TGCTCCGTATC
Bomo_Chr25	10368383	10368383	1	71	0	0	TGCTCCGTATC
Bomo_Chr25	10368407	10368407	1	75	0	0	TGCTCCGTATC

Bomo_Chr25	10368470	10368470	1	64	4	0.062	GCCAGCGCCAG
Bomo_Chr25	10368515	10368515	1	55	2	0.036	AGCTCCGCTTC
Bomo_Chr25	10368537	10368551	3	294	0	0	TCAGACGACCAAGCGTATTCGTAGC
Bomo_Chr25	10368575	10368575	1	37	4	0.108	ATATCCGCCAT
Bomo_Chr25	10368635	10368635	1	61	0	0	AGCACCGGCAC
Bomo_Chr25	10368659	10368659	1	66	1	0.015	AGCTCCGCTTC
Bomo_Chr25	10368677	10368677	1	71	2	0.028	TGCTCCGTATC
Bomo_Chr25	10368713	10368713	1	67	0	0	TGCTCCGTATC
Bomo_Chr25	10368737	10368737	1	71	1	0.014	TGCTCCGTATC
Bomo_Chr25	10368821	10368821	1	58	1	0.017	AGCACCGGCAC
Bomo_Chr25	10368893	10368893	1	42	0	0	AGCACCGGCTC
Bomo_Chr25	10368929	10368929	1	59	1	0.017	AGCTCCGCTTC
Bomo_Chr25	10368947	10368956	2	202	2	0.01	TGCTCCGTATCCAACGCCAG
Bomo_Chr25	10368971	10368971	1	78	0	0	TGCTCCGTATC
Bomo_Chr25	10368995	10368995	1	80	1	0.013	TGCTCCGTATC
Bomo_Chr25	10369019	10369019	1	77	1	0.013	TGCTCCGTATC
Bomo_Chr25	10369121	10369121	1	59	1	0.017	AGCACCGGCAC
Bomo_Chr25	10369157	10369157	1	71	0	0	TGCTCCGTATC
Bomo_Chr25	10369181	10369181	1	73	2	0.027	TGCTCCGTATC
Bomo_Chr25	10369295	10369301	2	178	0	0	TGAACCGGCACCGGCAC
Bomo_Chr25	10369319	10369319	1	64	2	0.031	AGCACCGGCAC
Bomo_Chr25	10369337	10369337	1	63	0	0	AGCACCGGCAC
Bomo_Chr25	10369355	10369355	1	58	1	0.017	AGCACCGGCAC
Bomo_Chr25	10369385	10369385	1	70	1	0.014	AGCTCCGCTTC
Bomo_Chr25	10369403	10369403	1	74	1	0.014	TGCTCCGTATC
Bomo_Chr25	10369415	10369415	1	74	1	0.014	TGCTCCGTATC

Bomo_Chr25	10369451	10369451	1	78	1	0.013	TGCTCCGTATC
Bomo_Chr25	10369475	10369475	1	75	0	0	TGCTCCGTATC
Bomo_Chr25	10369499	10369499	1	58	2	0.034	TGCTCCGGCTC
Bomo_Chr25	10369511	10369511	1	45	3	0.067	TGCTCCGGCTC
Bomo_Chr25	10369523	10369532	2	196	2	0.01	TGCTCCGTATCCAGCGCCAG
Bomo_Chr25	10369559	10369559	1	53	0	0	AGCACCGGCTC
Bomo_Chr25	10369577	10369577	1	63	0	0	AGCACCGGCAC
Bomo_Chr25	10369607	10369607	1	77	0	0	ACCTCCGTAAC
Bomo_Chr25	10369619	10369619	1	60	1	0.017	AGCTCCGGCAC
Bomo_Chr25	10369673	10369679	2	176	0	0	AGCTCCGGCACCGGCAC
Bomo_Chr25	10369697	10369697	1	46	5	0.109	AGCTCCGTAAC
Bomo_Chr25	10369715	10369715	1	55	0	0	AGCACCGGTAC
Bomo_Chr25	10369727	10369727	1	50	7	0.14	AGCACCGGCAC
Bomo_Chr25	10369748	10369748	1	78	0	0	ACCAGCGGCAG
Bomo_Chr25	10369793	10369793	1	59	3	0.051	AGCTCCGCTTC
Bomo_Chr25	10369811	10369820	2	186	0	0	TTGTCCGTACCCAGCGCCAG
Bomo_Chr25	10369859	10369872	3	309	0	0	AGCCCCGGCACCGGCACCCGCACC
Bomo_Chr25	10369895	10369895	1	60	0	0	AGCTCCGGCAC
Bomo_Chr25	10369931	10369931	1	65	2	0.031	AGCTCCGGCAC
Bomo_Chr25	10369967	10369967	1	58	0	0	AGCTCCGGCAC
Bomo_Chr25	10370003	10370003	1	70	2	0.029	AGCTCCGGCAC
Bomo_Chr25	10370039	10370039	1	64	2	0.031	AGCTCCGGCAC
Bomo_Chr25	10370075	10370075	1	66	1	0.015	AGCTCCGGCAC
Bomo_Chr25	10370093	10370093	1	55	2	0.036	AGCACCGGCAC
Bomo_Chr25	10370129	10370136	2	176	0	0	AGCTCCGGCACCCGCACC
Bomo_Chr25	10370150	10370150	1	77	2	0.026	ACCAGCGGCAG

Bomo_Chr25	10370177	10370177	1	74	1	0.014	TGCACCGACTG
Bomo_Chr25	10370201	10370205	2	208	2	0.01	GAAATCGCTCGTATA
Bomo_Chr25	10370243	10370252	2	210	0	0	TGATACGTATGGCCCGCTCT
Bomo_Chr25	10370271	10370271	1	76	0	0	GCTCCCGCATC
Bomo_Chr25	10370309	10370309	1	30	4	0.133	ACCATCGGAAA
Bomo_Chr25	10370351	10370367	4	408	0	0	GGACTCGTTACCGTCGGAATCCGTGGT
Bomo_Chr25	10370378	10370382	2	204	0	0	TATAACGAACGTCTT
Bomo_Chr25	10370434	10370434	1	43	4	0.093	TTTTCCGTTGC
Bomo_Chr25	10370465	10370465	1	71	3	0.042	TTCTTCGATAA
Bomo_Chr25	10370582	10370582	1	56	3	0.054	ATAAGCGACAT
Bomo_Chr25	10370627	10370627	1	73	5	0.068	CAATACGATAA
Bomo_Chr25	10370659	10370659	1	83	3	0.036	AATTTCGCACA
Bomo_Chr25	10370671	10370680	2	184	2	0.011	TCAATCGAAGTTAGCGCTGA
Bomo_Chr25	10370693	10370693	1	74	1	0.014	TGGAACGAGTT
Bomo_Chr25	10370753	10370753	1	81	3	0.037	TGGGGCGTTAG
Bomo_Chr25	10370827	10370827	1	59	2	0.034	CACCACGAGAT
Bomo_Chr25	10370872	10370872	1	75	0	0	TGTTTCGAGCT
Bomo_Chr25	10370918	10370923	2	196	12	0.061	AGCTACGCTCCGATGG
Bomo_Chr25	10371009	10371023	4	416	4	0.01	TATCACGTGATCGGTAACGCGACCC
Bomo_Chr25	10371044	10371044	1	75	2	0.027	TCTGGCGAGAA
Bomo_Chr25	10371065	10371065	1	83	1	0.012	CAGCTCGTGTT
Bomo_Chr25	10371114	10371114	1	93	3	0.032	TACCTCGAGGT
Bomo_Chr25	10371141	10371141	1	44	3	0.068	ATTTGCGAAAA
Bomo_Chr25	10371193	10371193	1	71	1	0.014	TAATGCGGTTA
Bomo_Chr25	10371219	10371219	1	72	4	0.056	TCTTTCGTCTA
Bomo_Chr25	10371243	10371243	1	53	3	0.057	CTTTACGAATT

Bomo_Chr25	10371297	10371297	1	34	4	0.118	TTTACCGGTGA
Bomo_Chr25	10371361	10371361	1	32	2	0.062	TTTGACGCAAA
Bomo_Chr25	10371426	10371426	1	85	0	0	TATTCCGTAGC
Bomo_Chr25	10371437	10371441	2	176	2	0.011	CCCCCCGGGCGTGAC
Bomo_Chr25	10371467	10371467	1	45	8	0.178	ATTTACGATAT
Bomo_Chr25	10371478	10371486	2	188	0	0	CTGTGCGTTTTGGCGACCA
Bomo_Chr25	10371509	10371517	2	196	0	0	TGGCCCGTGATATCGTCTG
Bomo_Chr25	10371568	10371568	1	102	3	0.029	CAGAGCGCAGC
Bomo_Chr25	10371617	10371617	1	87	2	0.023	GGAACCGAACT
Bomo_Chr25	10371667	10371677	2	196	4	0.02	ATTTTCGAGTTTTGACGTATT
Bomo_Chr25	10371689	10371689	1	78	2	0.026	ACTTTCGATAG
Bomo_Chr25	10371771	10371771	1	84	2	0.024	TACAACGTATG
Bomo_Chr25	10371873	10371873	1	81	3	0.037	AATATCGTTCA
Bomo_Chr25	10371932	10371940	2	146	0	0	AGGAACGGGACAGCGGAAA
Bomo_Chr25	10371986	10371989	2	152	10	0.066	CCAATCGGCGTGAT
Bomo_Chr25	10372159	10372159	1	68	3	0.044	TAGCACGACAT
Bomo_Chr25	10372233	10372233	1	37	1	0.027	TTTTACGGATT
Bomo_Chr25	10372259	10372267	2	212	4	0.019	TTTTCCGAAATATCGAAAA
Bomo_Chr25	10372305	10372305	1	83	1	0.012	CATCTCGTAAT
Bomo_Chr25	10372391	10372391	1	83	1	0.012	TTTTACGAAAA
Bomo_Chr25	10372424	10372424	1	84	1	0.012	CAGAACGATTT
Bomo_Chr25	10372436	10372438	2	166	2	0.012	GTTTTCGCGTAAC
Bomo_Chr25	10372470	10372470	1	2	0	0	AAATGCGTGTG
